# Zebrafish exposed to a cocktail of pesticides during early development display long-lasting neurobehavioral alterations

**DOI:** 10.1007/s00204-025-04129-6

**Published:** 2025-08-25

**Authors:** Maria Abellán-Álvaro, Isabel Forner-Piquer, Ieremias Chousidis, Elliott Godden, Alba García-Deante, Nicola Marchi, Caroline H. Brennan, Jose V. Torres-Pérez

**Affiliations:** 1 Department of Cellular Biology, Functional Biology and Physical Anthropology, University of Valencia, Burjassot, 46100 Valencia, Spain; 2 Unitat Predepartamental de Medicina, Universitat Jaume I, Castelló, Spain; 3 Department of Biomedical Sciences, Faculty of Health Sciences, Institute of Biomedical Sciences, Cardenal Herrera-CEU University, CEU Universities, Valencia, Spain; 4 Institute of Functional Genomics, CNRS, INSERM, University of Montpellier, Montpellier, France; 5 Department of Renewable Marine Resources, Marine Science Institute, Spanish National Research Council (ICM-CSIC), 08003 Barcelona, Spain; 6 School of Biological and Behavioural Sciences, Queen Mary University of London, London, E1 4NS UK

**Keywords:** Stress-reactivity, HPI axis, Critical period, Agrochemical, Pesticide, Early development

## Abstract

The widespread use of pesticides is increasing the presence of environmental contaminants with potential impacts on biodiversity, ecosystems, and human health. Although long-term pesticide effects have been previously studied, the long-term impact of an acute pesticide exposure during critical early developmental periods remains poorly understood. Here, we used zebrafish to examine whether acute exposure to a pesticide mixture at 0.5 μg/L (the maximum allowed in drinking water) during the first 5 days post-fertilisation (dpf) of development has lasting effects at 28 dpf. Zebrafish were assessed behaviourally, morphologically, and molecularly both immediately after exposure at 5 dpf and later at 28 dpf. Our results show alterations in stress-response that start to emerge right after the developmental exposure and are associated with a less anxious-like phenotype at juvenile stages. Interestingly, despite the observed behavioural phenotype at 28 dpf, it did not lead to significant molecular changes in the hypothalamic-pituitary-interrenal (HPI) axis at this stage. On the contrary, a positive control group of juvenile fish subjected to a sustained pesticide exposure throughout the 28 dpf showed both reduced anxiety-like behaviour and HPI alterations. Our study suggests that even an acute exposure to a low-concentration of pesticides during critical developmental periods can result in enduring behavioural changes.

**Supplementary Information:**

The online version contains supplementary material available at 10.1007/s00204-025-04129-6.

## Background

Pesticides are used worldwide to enhance crop yields and protect their production. The usage of pesticides has progressively increased their prevalence over time to approximately 3.6 million tonnes per year globally (Food and Agricultural Organization of the United Nations 2024), which has led to their presence in aquatic ecosystems and drinking water (Gilliom 2007; Van Bruggen et al. 2018). Pesticides are environmental contaminants significantly impacting biodiversity, ecosystem structure and function and human health (Zhou et al. 2025). The European Union Water Framework Directive has set a maximum standard of 0.5 μg/L for the total concentration of pesticides in drinking water (Directive 98/83/EC) and several directives regulate the presence of pesticide residues in food (i.e. No 396/2005, No 400/2014). 

Exposure to a single pesticide, or a mixture of various pesticides, has been associated with alterations in immune response, oxidative stress and neurological disorders, amongst others (Kamel and Hoppin 2004; Lee and Choi 2020; Forner-Piquer et al. 2021a, b; Sule et al. 2022; Jokanović et al. 2023). For instance, long-term exposure to pesticides has been shown to modify the hypothalamus–pituitary–adrenal (HPA) axis, leading to altered stress-responses (Michael Caudle 2016). Similarly, pesticides are known to possess endocrine-disrupting effects (Khoshnood 2024). However, the extent to which pesticide exposure during early development, a critical period of heightened susceptibility to environmental cues (Ventura et al. 2024), could result in long-term effects is poorly understood. Furthermore, studies on mixtures are often overlooked, assuming risks are negligible if individual components stay below safety limits (Kortenkamp et al. 2019).

Zebrafish (*Danio rerio*) make ideal candidates to study the effect of short- and long-term exposure to pesticides (Hill et al. 2005; Wang et al. 2017; Forner-Piquer et al. 2021b). Zebrafish have homologues for 70% of human genes (Howe et al. 2013; Bradford et al. 2017) and have a conserved neuroendocrine circuitry (Sheardown et al. 2022). In addition, previous studies have shown conserved effects of developmental exposure to pesticides on the neurodevelopment of the hypothalamus–pituitary–interrenal (HPI) axis, the zebrafish equivalent to the mammalian HPA axis (de Abreu et al. 2021).

Here, we used zebrafish to evaluate the long-term impact of an acute exposure to a low concentration of a mixture of pesticides during early development. In addition, we assessed whether this long-term effect of an acute exposure during early development was equally detrimental to a sustained exposure. Zebrafish were treated with a cocktail of commonly occurring agricultural pesticides similar to that previously used in zebrafish and rodents (Lukowicz et al. 2018; Forner-Piquer et al. 2021a, b). This mixture contains six pesticides, including ziram, captan, chlorpyrifos, thiacloprid, tiophanate and boscalid, but here we also added glyphosate (Forner-Piquer et al. 2021a). By incorporating a pesticide mixture, our study highlights the concept of mixture toxicity, which more closely resembles real-life environmental exposure scenarios.

## Material and methods

### Zebrafish husbandry

All animal procedures in this study were reviewed by the Queen Mary University of London (QMUL) ethics committee (AWERB) and conducted in accordance with the institutional guidelines for laboratory animal usage [European Union Council September 22, 2010 (2010/63)], Animals (Scientific Procedures) Act, 1986 and Home Office Licenses.

Adult Tübingen zebrafish (TU, wild-type strain) were kept under standardised conditions in a circulating system (Tecniplast, UK), with a photoperiod of 14 h light and 10-h darkness. The system uses aquarium treated water at 25–28 °C. Fish were fed twice daily, once with ZM-400 fry food or flakes (zmsystems) and once with live supplement.

Embryos were generated by natural spawning of two groups of approximately 50 zebrafish each bred in pairs using slope tanks. The males and females were separated overnight, divider removed the following day at 8.30 a.m. and eggs collected at 10.15 a.m. the same day. Embryos were reared in groups of 50 per Petri dish (100 mm × 20 mm, corning) with around 50 ml fish water (including, whenever required, the pesticide mixture; see Experiment 1 from Experimental design, below) in an incubator under standard conditions until 5 days post-fertilisation (dpf). Following day (at 6 dpf) larvae were transferred to tanks and reared until 28 dpf in the presence/absence of the pesticide mixture (see Experiment 2 from Experimental design, below) under similar conditions as adult fish.

### Drug administration and zebrafish maintenance

The compounds used were a cocktail, hereafter referred to as PEST, of 6 target pesticides (ziram, captan, tiophanate, thiacloprid, chlorpyrifos, boscalid,) at similar concentrations as used previously (Forner-Piquer et al. 2021a) plus glyphosate (Forner-Piquer et al. 2021a). PEST working concentration was determined at 0.5 μg/L for total pesticides, based on the maximum concentration stipulated by the European drinking water (98/83/EC) and the ground water (2006/118/EC) directives.

Pesticides were diluted in fish water to a final concentration of 0.07 μg/L for an individual pesticide and 0.5 μg/L for total pesticides. Dimethyl sulfoxide (DMSO) was used to prepare a PEST stock solution at 0.5 mg/mL. Therefore, since the final working concentration of DMSO in the fish water was below 0.01%, a concentration deemed as safe for zebrafish developmental toxicity assays (Forner-Piquer et al. 2021b; Hoyberghs et al. 2021), sham controls (DMSO-treated fish) were not included. Stock solution aliquots were stored at − 20 °C and thawed as needed.

Approximately 80% of the fish water from water-treated and experimental groups were renewed daily. Each experimental rearing condition was performed in duplicate to avoid a possible ‘tank effect’. Animals from both experiments came from the same breeding batch.

### Experimental design

**Fig. 1:**
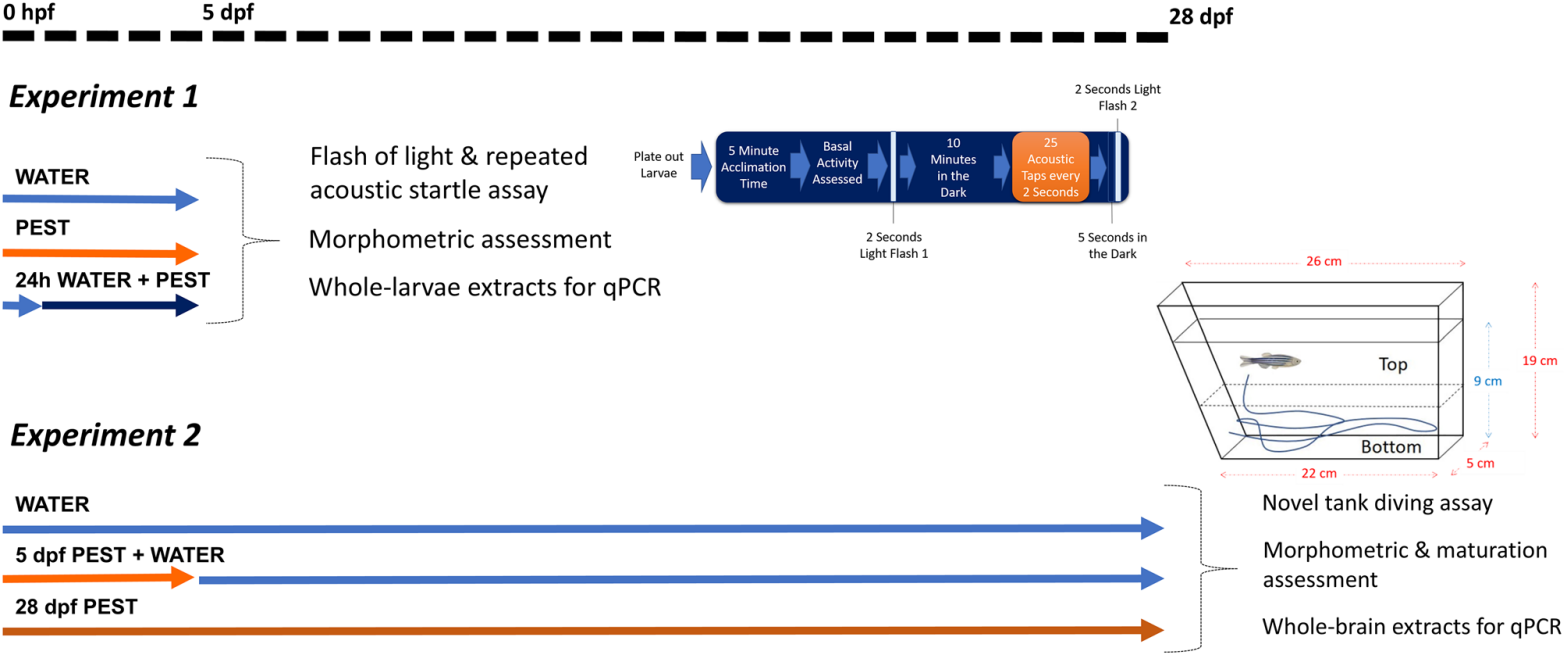
Experimental design. Zebrafish eggs resulting from a massive simultaneous breeding were combined and randomly allocated in groups of 50 and exposed to different treatments (with at least two replicates per group). Same breeding stock was used to conduct both Experiment 1 and Experiment 2. For Experiment 1, embryos were treated to a mix of pesticides from 2 hours post-fertilisation (hpf) to 5 days post-fertilisation (dpf) (PEST); control groups include negative control without PEST (WATER) and embryos exposed from 1 to 5 dpf to PEST (24hWATER + PEST) to assess the effect of PEST after the neuromuscular junction has been formed. After the 5-day exposure, some larvae were subjected to a behavioural assay consisting of a 2 seconds (s) flash of light followed by a repeated acoustic stimulus and an additional 2 s flash of light (see detailed protocol including the timings of each stage in the figure’s diagram or Material and Methods section). Finally, some larvae were collected for either morphological assessment or RT-qPCR experiments. Remaining 5 dpf larvae from Experiment 1 were consequently reared for Experiment 2: embryos previously exposed to PEST from 2 hpf to 5 dpf and were then transferred to fresh clean water up to 28 dpf (5dpfPEST + WATER); control groups included negative control (WATER) and fish exposed to PEST throughout the whole experimental time (28dpfPEST, positive control). At the end of corresponding exposure, juvenile fish were individually subjected to the novel tank diving test (NTT, a trapezoid-shaped tank with 19 cm height, 5 cm width, 26 cm top length, and 22 cm bottom length; containing 1 L of system water with 9 cm water height). Finally, some fish were collected for morphological and maturation assessment and the brains extracted to conduct RT-qPCR experiments. Above dashed line indicates a temporal line. Note that behavioural assays are not scaled

This study consists of two interconnected experiments (Fig. 1) derived from a single rearing phase, ensuring consistency across developmental conditions. Experiment 1 (see below) focuses on assessing the immediate effects of early-life acute pesticide exposure at 5 dpf by evaluating behavioural, morphological, and molecular endpoints. A subset of larvae from each condition was collected at this time point for these analyses. The remaining larvae from the same rearing conditions were then maintained under controlled conditions until 28 dpf to assess the long-term effects of this early-life exposure (Experiment 2, see further details below).

#### Experiment 1

This experiment aims to investigate potential stress-related differences due to an acute exposure to 0.5 μg/L of PEST during the first 5 days of the fish, a critical period characterised by increased developmental plasticity, which can be highly influenced by environmental cues, and encompassing rapid and fundamental maturation of the nervous system and other physiological systems.

Following a multi-pair breeding event, all eggs were pooled together, rinsed in fresh fish water, sorted fertile from infertile and randomly divided into groups of 50 according to treatment conditions. Treatment/Experimental conditions (at least four dishes each) were as follow: (1) 5 dpf + water (group referred to as WATER): Negative control; (2) 5 dpf + PEST (PEST): Exposure to PEST started at around 2 h post-fertilisation (hpf) until 5 dpf; (3) 24 h water + PEST (24hWATER + PEST): Exposed to water for the initial 24 hpf followed by PEST for the remaining 4 days. Condition (3) was included to differentiate between stress-related behaviours and distorted neuro-muscular development as neuro-muscular junction starts to develop at 24 hpf (Fichi et al. 2019).

At 5 dpf, a proportion of animals from all conditions were subjected to a behavioural assay (see below). Then, some animals were fixed for morphological evaluation and others snap-frozen for subsequent RT-qPCR analysis (see below). In addition, mortality rate was determined across the 5 day period. The remaining 5 dpf fish not used for behavioural and molecular analysis were continued for Experiment 2. Experimenter was blind to treatment/condition.

#### Experiment 2

This experiment aims to assess the long-term effect of an early-life acute exposure of 0.5 µg/L of PEST. We chose 28 dpf as this age represents the transition from larval to juvenile stage, coinciding with the beginning of sex differentiation, pigment and scale development and emergence of complex behaviours (Singleman and Holtzman 2014; King et al. 2020).

Remaining animals from Experiment 1 from the conditions WATER and PEST were transferred to tanks where they were reared until 28 dpf in the presence/absence of PEST to obtain the following experimental conditions: (1) 28 dpf + water (WATER): Negative control group; (2) 5 dpf PEST + water (5dpfPEST + WATER): Exposed to PEST from 2 hpf to 5 dpf and then to water for 23 days until 28 dpf; (3) 28 dpf PEST (28dpfPEST): Exposed to PEST from 2 hpf to 28 dpf. The condition (3) was included as a positive control, to rule out differences between early life effects from those of chronically exposed fish.

On the 28th day, fish were subjected to a behavioural assay. Following, some brains were extracted for RT-qPCR, and some fish fixed to assess morphology and maturation state. Experimenter was blind to treatment/condition.

### Behavioural assays

#### Experiment 1: the flash of light and acoustic startle assay

This protocol (Fig. 1) is a combined assay that relies on the fish response to sudden change in environmental illumination, previously associated with stress-reactivity (Lee et al. 2019; García‐González et al. 2021; Faught and Vijayan 2022), and their habituation to acoustic cues, used to assess associative learning (Tegelenbosch et al. 2012; García‐González et al. 2021; Beppi et al. 2021) and measure hyper-arousal associated with exaggerated stress responses (Cohen et al. 2006; Tanaka et al. 2019).

At 5 dpf, zebrafish were subjected to a behavioural test we used in the past that combines a light-locomotor behavioural assay, and a startle tap test habituation assay (Torres-Pérez et al. 2023) with small variations (Fig. 1). Briefly, zebrafish had an acclimation period inside a dark DanioVision Observation Chamber (Noldus Information Technology, Wageningen, The Netherlands) of 5 min (time not recorded). Following the acclimation period, recording started. First, their basal activity was assessed during one minute in the dark. Then, there were 2 s of stimulation with white light and back to dark for 9 min and 58 s. Next, there were 25 consecutive sound/vibration stimuli with an inter-stimulus interval of 2 s. Finally, there was another stimulation with white light for 2 s and back to the dark for 5 s after which the protocol finished. EthoVision software (Noldus Information Technology, Wageningen, NL) was used to track larval movement. Data were output into one second time-bins.

#### Experiment 2: novel tank diving assay (NTT)

This assay, widely used to determine anxiety-driven responses in adult zebrafish (Maximino et al. 2010; Evans et al. 2021), was adapted for 28 dpf juvenile zebrafish and the exposure conditions (Fig. 1). First, fish from all experimental groups were transferred to untreated water for a 5 min period to allow for acclimation to the new temperature and to ensure no PEST contaminated their new environment (in the case of 28dpfPEST). Zebrafish were then individually tested in a 1 L trapezoid-shaped tank with dimensions 19 cm height × 5 cm width × 26 cm top length × 22 cm bottom length, filled with a litre of fish water (water replaced every 2–3 trials) to a maximum depth of 9 cm. Fish behaviour was side view recorded for 6 min using a DMK 21AF04 Firewire Monochrome camera. EthoVision software (Noldus Information Technology, Wageningen, NL) was used to track and analyse behavioural swimming activity including the parameters of bottom dwelling, mobility state and distance covered.

### Morphological evaluation

#### Experiment 1

Following behavioural assay, 8 randomly selected fish from each condition (4 from each experimental duplicate) were fixed overnight in 4% paraformaldehyde (PFA) in a phosphate-buffered solution (PBS, pH = 7.4). Following day, fish were transferred to fresh PBS and two pictures taken for each fish with a Leica EZ4HD stereo microscope coupled to a digital camera (Leica DFC495, Wetzlar, Germany), one providing a lateral view and the other presenting the dorsal portion, to assess morphological aspects, as adapted from Martínez et al. (Martínez et al. 2019). Measurements (Sup. Figure 1) were performed with the software ImageJ (imagej.nih.gov/ij/). From the lateral-view images: Body length (BL). From the dorsal-view images: Eye-Snout Distance (ESD), Head Width (HW), Inter-Ocular Distance (IOD), Eye Width (EW; both left and right), and Eye Length (EL; both left and right). Although there were no differences in BL (see results), the additional morphological measures were normalised to each fish’s BL to ensure scalability of the measures, similarly to our previous research (Torres-Pérez et al. 2023).

#### Experiment 2

As with experiment 1, selected juvenile fish were fixed with PFA overnight and then transferred into PBS to take pictures and assess morphological aspects. Measurements included in analysis are: ESD, HW, IOD, EW, EL and BL. In addition, the maturation stage at 28 dpf was also assessed based on the phenotypically rating of pigmentation at skin, tail fin, anal fin and dorsal fin (Singleman and Holtzman 2014).

### Real time quantitative PCR (RT-qPCR)

#### Experiment 1

Following corresponding behavioural assay, randomly selected 5 dpf zebrafish larvae were pooled in groups of 8 individuals from all corresponding conditions (4 from each experimental duplicate), snap-frozen in liquid nitrogen and stored at − 80 °C until use. RNA extraction, cDNA synthesis and RT-qPCR were performed and validated as previously reported (Torres-Pérez et al. 2023). Briefly, we used SYBR Green and the CFX Connect Real-Time System, with all reactions performed in triplicate. The protocol included an initial denaturation at 95 °C for 5 min, followed by 50 cycles of 95 °C for 10 s, 60 °C for 12 s, and 72 °C for 12 s. Relative mRNA expression was determined using a modified Pffafl method, accounting for multiple reference genes and minor variations in primer amplification efficiency (Evans et al. 2021). β-Actin (*actb2*) and ribosomal protein L13a (*rpl13α*) were used as housekeeping genes to normalise the data and to assess relative gene expression changes. Full list of primers, including accession number, sequence, product size, efficiency, and source, can be found in Supplementary Table 1. Like our previous study (Evans et al. 2021), we also included the ratio mineralocorticoid receptor / glucocorticoid receptor alpha (*mr/grα)* as a marker of HPI reactivity.

#### Experiment 2

After NTT, randomly selected 28-dpf zebrafish were culled with an overdose of tricaine (MS-222) to extract their brain, which were snap-frozen and kept at − 80 °C until use. Similar RT-qPCR as in Experiment 1 were performed with these brains.

### Statistical analysis

Except behavioural data, all experiments were analysed and graph performed using GraphPad Prism 9.0.2 for Windows (GraphPad Software, San Diego, California USA, www.graphpad.com). Normal distribution and homoscedasticity were visually assessed with QQ plots. A mortality rate between conditions over the first 5 days was analysed with a mixed-effects model with the Geisser-Greenhouse correction with Tukey’s multiple comparisons. Morphological analysis (both at 5 and 28 dpf) and maturation scores were analysed as ordinary one-way ANOVA with Tukey’s multiples comparison if passed normality test for at least three of the following tests: D’Agostino-Pearson omnibus, Anderson–Darling, Shapiro–Wilk and Kolmogorov–Smirnov. If a morphological measure did not pass normality, a Kruskal–Wallis test with Dunn’s multiple comparison test was used (significance level at p < 0.05, Bonferroni correction). Data from RT-qPCR analysis was analysed using an ordinary two-way ANOVA with ‘genotype’ (either genes related to the HPI axis, to oxidative stress, or to previously related with pesticide exposure) and “time” as factors and ‘subject’ as matched set and using Bonferroni’s multiple comparison test. All effects are reported significant at p < 0.05.

All statistical analyses for behavioural experiments were conducted using the program R (Team 2023). All statistical tests adhered to a significance level of p < 0.05. Mixed linear models (GLMM: Gamma distribution with log-link) were primarily employed to account for both fixed effects (e.g. treatment) and random effects (e.g. individual fish variability), unless stated otherwise. Outliers were identified and removed when they represented extreme deviations, as determined by statistical tests (e.g. residual analysis with studentized residuals) to ensure robustness and improve the stability of the model.

To ensure compatibility with the GLMM, proportional adjustments were applied to the data to avoid non-positive values. This transformation preserved the relative relationships between observations whilst meeting the model’s assumptions (Harrison et al. 2018). Model selection was based on evaluating model convergence, goodness-of-fit metrics such as Akaike Information Criterion (AIC), and the theoretical consistency of model terms with the experimental design. Assumptions of normality and homogeneity of variances were assessed, prior to fitting models, using Kolmogorov–Smirnov and Levene’s test respectively.

Beta regressions were employed for response variables expressed as proportions (e.g. percentage of responders), as it is specifically designed for continuous bounded data between 0 and 1. This approach was chosen due to the non-normal distribution of the data and its suitability for flexible modelling using link functions appropriate for proportions. In addition, gamma-distributed GLMMs were used for non-proportional data (e.g. total movement) to account for positively skewed distributions.

The behavioural data visualisation was performed using Python 3.12.3 and the following libraries: Matplotlib (v3.10.0), Seaborn (v0.13.2), Pandas (v2.2.3), and NumPy (v2.2.1).

## Results

Zebrafish were used to assess whether an acute exposure to a low concentration of pesticides during early development had lasting effects. For that aim, experimental procedures were divided in two experiments (Fig. 1), both emerging form a single breeding event.

The concentration of PEST at which subjects were exposed, 0.5 μg/L, the maximum permitted concentration for total pesticides in drinking water, did not lead to an increased mortality of eggs/embryos during the first 5 dpf, as no significant differences between treatments were observed (Sup. Figure 2).

**Fig. 2:**
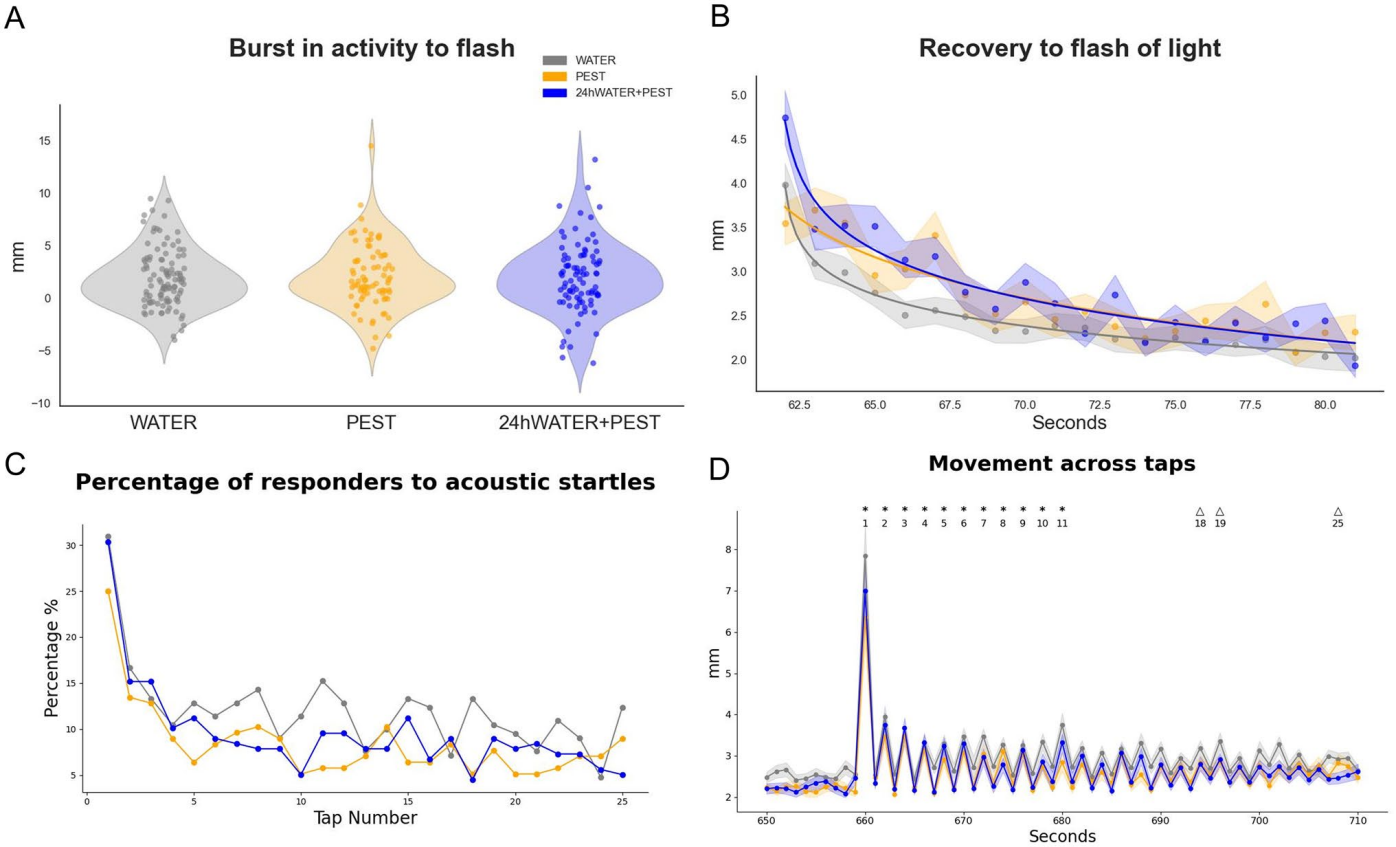
Behavioural response to the ‘Flash of Light and Acoustic Startle Assay’ for 5 dpf zebrafish from Experiment 1. **A** Burst in activity (in mm) in response to the first flash of light for WATER (grey), PEST (orange) and 24hWATER + PEST (blue) groups; violin plots include points as single values. **B** Rate of habituation (R^2^ for each group as coloured line) from the mean distance moved (mm) to the 20 s following the first flash of light. **C** Percentage of fish responding to the 25 consecutive taps (cues for acoustic startle). **D** Mean distance moved by the fish of each group during the taps (acoustic cues). **B** and **D** show mean with ± Standard Error Mean (SEM) as shadows. *p < 0.05 for WATER *vs* PEST at corresponding tap number; Δp < 0.05 for WATER *vs* 24hWATER + PEST at corresponding tap number (annotated below each symbol)

### Zebrafish larvae exposed to PEST show increased stress-reactivity at 5 dpf

To assess differences in basal locomotor activity, we measured total movement (distance travelled) for each fish during the first recorded minute (Basal 1 period). The comparison did not reveal any significant effect of treatment on basal locomotor activity (χ^2^ = 1.0085, p = 0.604). Compared to WATER-treated group, neither the 24hWATER + PEST nor the PEST groups showed significant difference (β = − 0.0019, p = 0.981; β = − 0.0738, p = 0.362; respectively). We similarly assessed total movement (distance travelled) for each fish during the four minutes following the initial 60 s after the flash (Basal 2 period: seconds ≥ 122 & seconds ≤ 362). As with Basal 1, there were no significant differences (χ^2^ = 0.3497, p = 0.8396; WATER *vs* 24hWATER + PEST: β = − 0.0260, p = 0.658; WATER *vs* PEST: β = − 0.0333, p = 0.584). Therefore, no pre-existing differences in movement were observed across the groups.

Next, we analysed the zebrafish reaction to the first flash of light, as this behaviour is mediated via their immediate stress response (Lee et al. 2019). First, we assessed the locomotor ‘burst activity’ in response to the change in illumination by comparing the movement of the fish before and after the flash (Fig. 2A). Normality tests on the data without outliers indicated a normal distribution (Kolmogorov–Smirnov D = 0.0615, p = 0.257). In addition, Levene’s test confirmed homogeneity of variances across treatment groups (F_(2,268)_ = 0.879, p = 0.416). Based on these results, a linear model (LM) was deemed appropriate to analyse the ‘burst activity’. This comparison did not reveal significant differences between groups (R^2^ = 0.0002, F_(2,268)_ = 0.031, p = 0.970).

Second, to evaluate the rate of recovery after the first flash of light we analysed movement over a 20 s period after the flash (starting at second 62, referred to as the 1st second, through second 81, the 20th second; Fig. 2B) using a GLMM with a Gamma distribution and log link. This analysis showed a significant effect of ‘time’ when comparing both conditions, 24hWATER + PEST and PEST, to WATER (β = − 0.0346, p < 2 × 10^−16^), indicating a general decrease in movement as time progressed. When assessing the effect of ‘treatment’ we observed that the 24hWATER + PEST group differed significantly from WATER (β = 0.6819, p = 0.00114) whilst PEST groups did not (β = − 0.0168, p = 0.93842). Similarly, when using 24hWATER + PEST as the reference group, a significant ‘treatment’ effect was observed in comparison to PEST (β = − 0.6987, p = 0.00201).

In addition, the interaction between ‘treatment’ and ‘time’ indicated slower recovery rate for the 24hWATER + PEST group compared to both WATER (β = − 0.0081, p = 0.00323) and PEST (β = − 0.0088, p = 0.00292) groups, by which 24hWATER + PEST maintained a higher level of movement throughout the recovery period. PEST group did not differ significantly from the WATER group in either locomotor activity (p = 0.938) or recovery rate (TreatmentPEST x Time (seconds): p = 0.253). Nonetheless, *post-hoc* comparisons of their rate of recovery did not reveal statistically significant differences in locomotor activity between groups (seconds 62–76, *p* > 0.05).

We then assessed the habituation of the fish to the acoustic startle stimulation in terms of both percentage of fish responding to each tap (Fig. 2C) and mean distance moved at each tap (Fig. 2D). All groups demonstrated habituation when considering the percentage of fish reacting to each tap across ‘treatment’ groups (WATER, PEST, and 24hWATER + PEST), with a significant decrease (β = − 0.0437, p < 0.0001) in the percentage of responders over time, from the 1st (WATER: 55.24%, PEST: 48.72%, 24hWATER + PEST: 53.93%) to the 25th tap (WATER: 20.00%, PEST: 16.67%, 24hWATER + PEST: 10.11%). There were no significant differences between groups (p > 0.05).

The GLMM analysis revealed that the interaction between ‘treatment’ and ‘tap number’ was not statistically significant (p > 0.1 for all interaction terms). However, ‘tap number’ had a significant main effect on ‘probability of response’ (β = − 0.057, p < 0.001), confirming that fish habituated to repeated stimuli. Estimated slopes for each treatment were as follows: 24hWATER + PEST (− 0.0571 ± 0.0077), WATER (− 0.0411 ± 0.0064), and PEST (− 0.0473 ± 0.0085), with no significant pairwise differences between treatments (Tukey-adjusted p > 0.2 for all comparisons).

The GLMM to analyse the total movement of zebrafish across the 25 taps (Fig. 2D), excluding the inter-tap movement, revealed a significant habituation effects over ‘time’ (tap.number, p < 0.001). Fish in the WATER group showed higher initial movement levels compared to the other groups, with a significant difference between the WATER and 24hWATER + PEST groups (β = 0.1083, p = 0.0438) and a significant difference between WATER and PEST (β = 0.1413, p = 0.0112). The interaction between treatment and tap number was not significant (WATER × tap.number: β = 0.0001, p = 0.921; PEST × tap.number: β = 0.0018, p = 0.243). *Post-hoc* pairwise comparisons of the total movement of fish after each tap showed that WATER group moved significantly more than the PEST group during the early taps (1 to 11, Fig. 2D), but these differences faded in the later taps.

As with the previous flash of light, no significant differences were observed between treatment groups in their immediate reaction to the second flash of light. The estimated effect for WATER relative to 24hWATER + PEST was not significant (Estimate = 0.0209, p = 0.540), and the effect for PEST relative to 24hWATER + PEST was also not significant (Estimate = − 0.0388, p = 0.291).

### PEST exposure alters morphology and the expression of HPI genes at 5 dpf

**Fig. 3:**
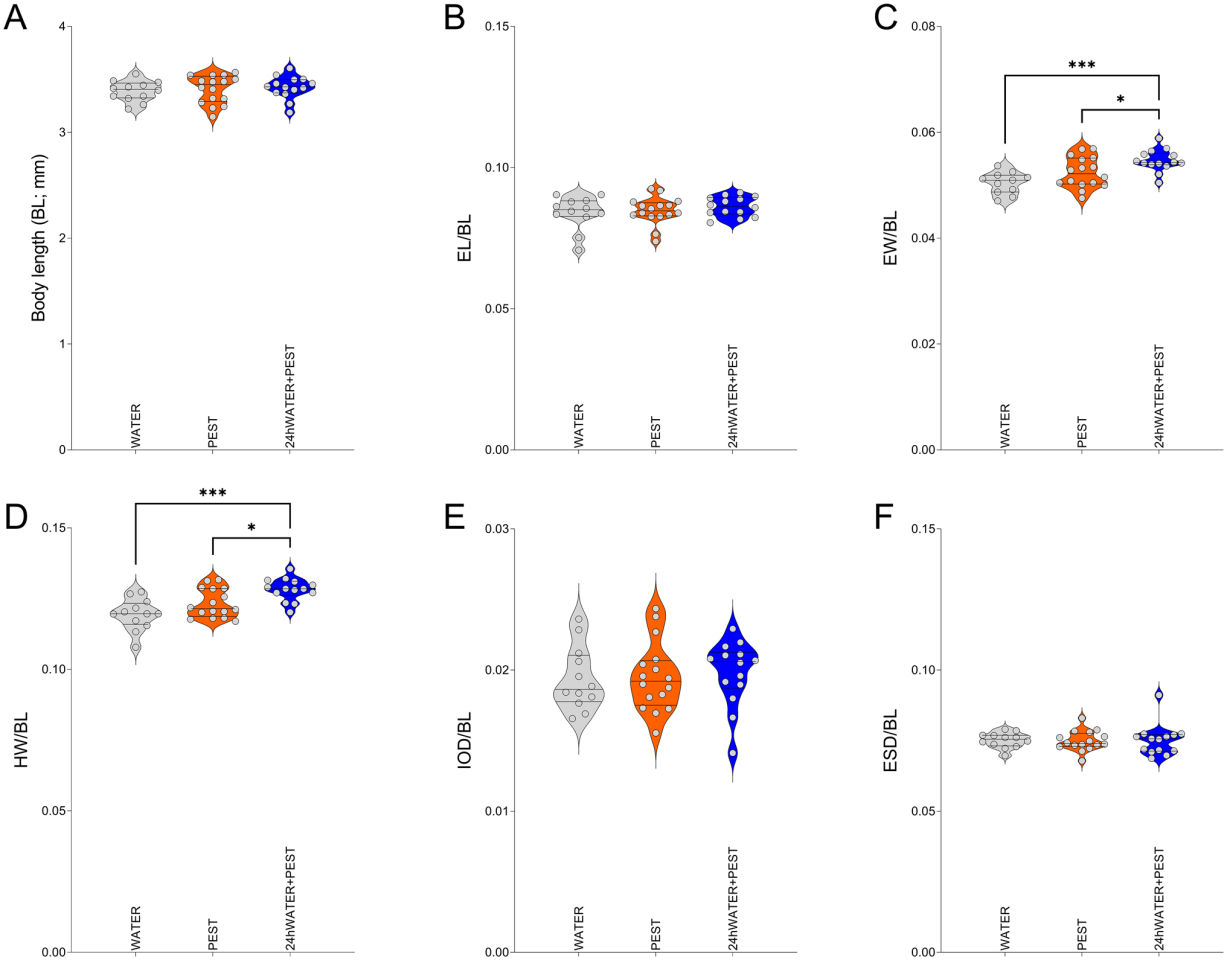
Morphological assessment for 5 dpf fish from Experiment 1. Morphological assessment of fixed larvae following behavioural assessment included the following measures: (**A**) total body length (BL), (**B**) eye length (EL), (**C**) eye width (EW), (**D**) head width (HW), (**E**) inter-ocular distance (IOD), (**F**) eye-snout distance (ESD). All measures corrected by BL, except BL itself. Violin plots include points as single values. *p < 0.05, ***p < 0.001 *vs* corresponding contrast

**Fig. 4:**
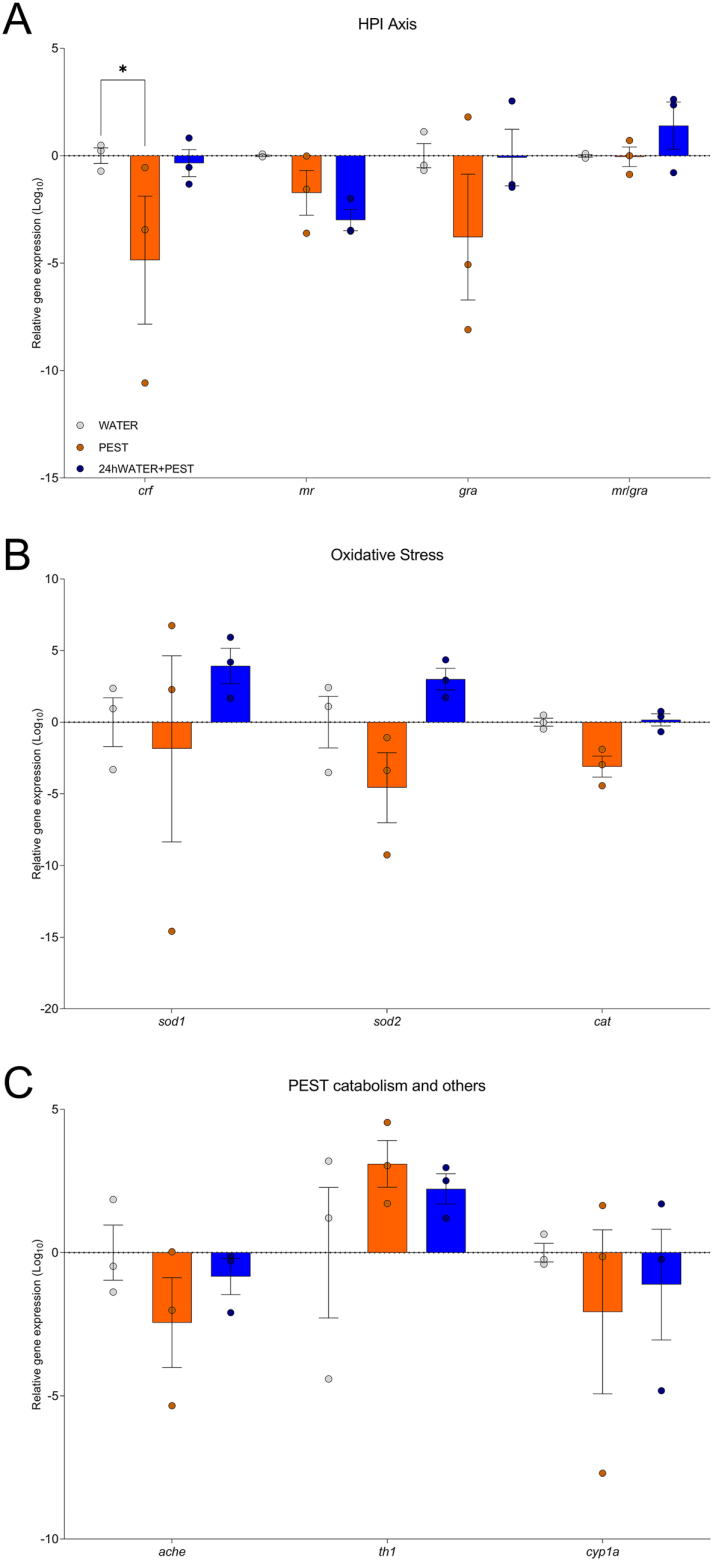
RT-qPCR profiling for 5-dpf fish from Experiment 1. **A** RT-qPCR analysis for genes related to the HPI axis including *crf* (codifying corticotropin releasing factor), *mr* (mineralocorticoid receptor), *grα* (glucocorticoid receptor α) and the ratio *mr/grα*. **B** RT-qPCR for genes associated with an oxidative stress response, including *sod1* and *sod2* (superoxide dismutase 1 and 2), and *cat* (catalase). **C** RT-qPCR for genes involved in the catabolism of PEST and other genes previously associated with pesticide exposure, including *ache* (acetylcholinesterase), *th1* (tyrosine hydroxylase 1) and *cyp1a* (cyto-chrome P4501A family 1 subfamily **A**). Graphs show mean ± SEM, dots show individual values. n = 3 pools of 8 embryos each; *p < 0.05 *vs* corresponding contrast

Following behavioural evaluation at 5 dpf (Experiment 1), randomly selected fish from each treatment were preserved for morphological assessment (Fig. 3, n = 12–16 per group) whilst others were snap-frozen and processed for RT-qPCR analysis (Fig. 4, n = 3, each replicate consisting of a pool of 8 fish).

Morphological analysis (Fig. 3, Sup. Figure 1) showed that both eye width (EW) and head width (HW) were significantly affected by treatment (Fig. 3A, EW: F (2, 38) = 9.500, p = 0.0005; Fig. 3D, HW: F (2, 39) = 10.74, p = 0.0002). *Post-hoc* analysis demonstrated that this effect was due to a significant increase of both EW (p = 0.0003) and HW (p = 0.0001) in the 24hWATER + PEST fish when compared to water-treated animals, whilst subjects treated with PEST from 2 hpf did not differ in these measures to water-treated controls (EW: WATER *vs* PEST: p = 0.1025, PEST *vs* 24hWATER + PEST: p = 0.417; HW: WATER vs PEST: p = 0.0983, PEST vs 24hWATER + PEST: p = 0.0247). No other significant differences were detected (p > 0.05).

The expression analysis of genes implicated in the HPI axis (Fig. 4A) revealed a significant effect of ‘treatment’ (F (2, 24) = 4.018, p = 0.0312) but not of ‘gene of interest’ (F (3, 24) = 1.599, p = 0.2157) or the interaction between both factors [F (6, 24) = 1.289, p = 0.2995]. *Post-hoc* comparison showed that this effect was due to a significant difference between WATER and PEST treated fish for the *crf* gene (p 0.0399; other comparisons: p > 0.05), indicating a diminished activation of the HPI axis in PEST fish. Treatment also had a significant effect on the expression of genes involved on oxidative stress (Fig. 4B; F (2, 18) = 3.648, p = 0.0468), although *post-hoc* comparisons did not reveal any significant pairwise differences (p > 0.05). Finally, the expression of genes involved in pesticide metabolism and associated with pesticide-induced neurodegeneration (Fig. 4C), were not significantly affected (p > 0.05).

### Early-life acute exposure to PEST reduces anxiety-like behaviours in juvenile zebrafish (28 dpf) similarly as a sustained exposure

**Fig. 5:**
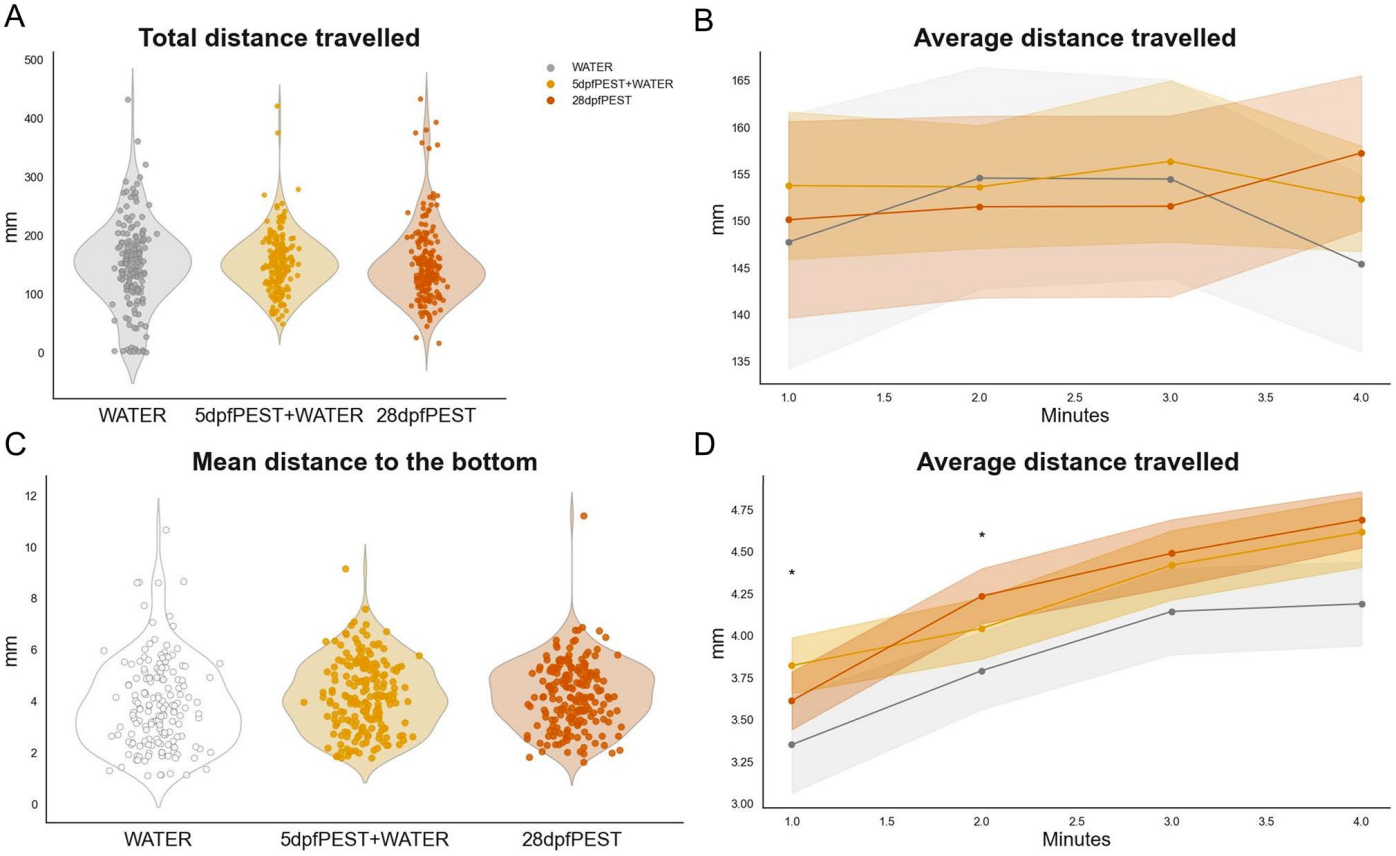
Behavioural response to the ‘Novel Tank Diving Test’ (NTT) for 28 dpf fish from Experiment 2. **A** Total distance (in mm) travelled by the fish during their time at the NTT for WATER (grey), 5dpfPEST + WATER (yellow) and 28dpfPEST (dark orange) groups. **B** Average distance travelled (mm) per minute in the assay. **C** Mean distance (in mm) to the bottom of the tank. **D** Average distance (in mm) to the bottom per minute in the assay. Violin plots (**A** and **C**) include points as single values. Graphs (**B**) and (**D**) show line as mean value with ± SEM as shadows. *p < 0.05 for WATER *vs* 5dpfPEST + WATER at corresponding timepoint

First, we assessed the total distance travelled by the fish (Fig. 5A, B), which served to measure potential differences on the overall activity induced by treatment. Our statistical model suggested that neither factor ‘time’ (β = − 0.0000051, p = 0.993) nor ‘treatment’ (β_5dpfPEST+WATER_ = 0.0434, p = 0.745; β_28dpfPEST_ = − 0.0410, p = 0.756) had a significant effect on the total distance travelled by the fish. Similarly, the interaction (how distance changes over time for different treatments) was also non-significant (β_time:5dpfPEST+WATER_ = 0.0000017, p = 0.998; β_time:28dpfPEST_ = 0.000319, p = 0.665).

Second, we investigated the mean distance to the bottom of the tank through time in the NTT (Fig. 5C, D). This measure indicates how far the fish stayed from the bottom of the tank over time, providing insights into its anxiety levels. There were significant differences of both ‘Distance to Zone’ (zone being the ‘bottom of the tank’) across treatment groups and time (β = 0.0017, p < 0.001), thus suggesting there was a reduction in anxiety-like response throughout the time in the new environment.

Specifically, the WATER-treated group spent significantly more time at the bottom of the tank compared to both the 5dpfPEST + WATER group (β = 0.231, p = 0.013) and the 28dpfPEST group (β = 0.151, p = 0.088), indicating a potential anxiolytic effect of PEST exposure. However, there were no significant differences between the 5dpfPEST + WATER and 28dpfPEST groups (interaction term seconds: 5dpfPEST + WATER, β = − 0.0006, p = 0.162; seconds: 28dpfPEST, β = − 0.0003, p = 0.567).

When analysing *post-hoc* results by time intervals (minute bins), the mean distance to the bottom varied across treatments and time points, reflecting dynamic anxiety-like behaviours over the course of the test. Significant differences between the WATER-treated group and the 5dpfPEST + WATER group were observed in the earlier stages of the test by which the WATER group spent more time close to the bottom of the tank (1st minute: p = 0.027; 2nd minute: p = 0.0413), thus indicating lower anxiety-like responses in the PEST-exposed group during these initial time intervals. At later time points (180 and 240 s), these differences were no longer significant. Comparisons between 28dpfPEST and WATER groups and between 5dpfPEST + WATER and 28dpfPEST groups did not yield significant differences at any time point (p > 0.05).

### PEST exposure exacerbates HPI axis alterations by 28 dpf

**Fig. 6:**
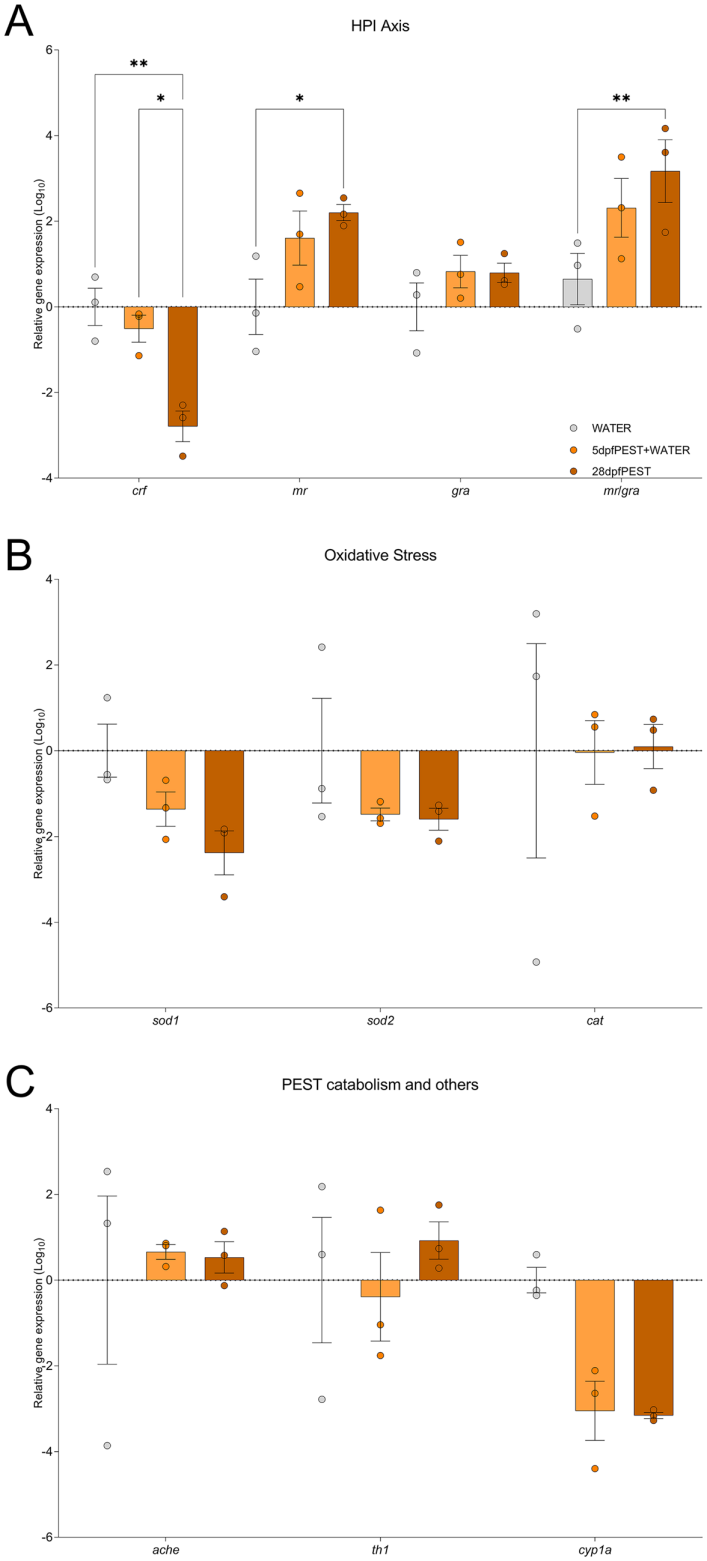
RT-qPCR profiling for 28 dpf fish from Experiment 2. **A** RT-qPCR analysis for genes related to the HPI axis: *crf*, *mr*, *grα* and the ratio *mr/grα*. **B** RT-qPCR for genes associated with an oxidative stress response: *sod1* and *sod2*, and *cat*. **C** qPCR for genes involved in pesticide catabolism: *ache*, *th1* and *cyp1a*. Graphs show mean ± SEM, dots show individual values. n = 3 pools of 3-5 brains each: *p < 0.05 *vs* corresponding contrast. *p < 0.05, **p < 0.01 *vs* corresponding contrast

All juvenile zebrafish at 28 dpf were culled after NTT (Experiment 2). Some of those fish were randomly selected for morphological assessment (Sup. Table 2 and Sup. Figure 3, n = 8 per treatment group) whilst the brains of others (also randomly selected) were extracted and snap-frozen for RT-qPCR (Fig. 6, n = 3 per replicate, each replicate consisting of a pool of 3-5 brains).

Statistical analysis did not reveal any significant differences (all p > 0.05) in terms of overall maturity score (Sup. Table 2) nor in morphometric measures (Sup. Figure 3). Therefore, this suggests that neither PEST treatment induced any major morphological or maturation change in the fish at 28 dpf.

On the other hand, statistical analysis of genes implicated in the HPI axis by RT-qPCR (Fig. 6A) indicated a significant effect of ‘gene of interest’ [F (3, 24) = 20.60, p < 0.0001] and a close-to-significant effect of ‘treatment’ [F (2, 24) = 3.344, p = 0.0523]. In addition, the interaction ‘gene of interest x treatment’ was significant [F (6, 24) = 5.720, p = 0.0008]. *Post-hoc* comparisons demonstrated a significant decrease in the expression of *crf* in the 28dpfPEST when compared to both WATER (p = 0.0015) and 5dpfPEST + WATER groups (p = 0.0130), and a significant increased expression of *mr*, and in the *mr/grα* ratio, when compared to WATER group (p = 0.0168 and p = 0.0057, respectively) but not to the 5dpfPEST + WATER group (p = 0.1084 and p = 0.0913, respectively). No other comparison for the HPI-related genes was significant (p > 0.05). Thus, our data points to a dysregulation in the HPI axis induced by the exposure to PEST.

Genes coding for oxidative stress markers (Fig. 6B) did not show any effect of ‘treatment’ [F (2, 18) = 1.287, p = 0.3003], ‘gene of interest’ [F (2, 18) = 1.303, p = 0.2961], or the interaction of both factors [F (4, 18) = 0.4140, p = 0.7963].

Finally, genes associated with pesticide metabolism (Fig. 6C) only showed a significant effect of ‘gene of interest’ (‘gene of interest’: F (2, 18) = 6.303, p = 0.0084; ‘treatment’: F (2, 18) = 0.7392, p = 0.4914; interaction: F (4, 18) = 1.768, p = 0.1792). However, no *post-hoc* comparison was significantly different (all p > 0.05).

## Discussion

We exposed zebrafish from 2 hpf to 5 dpf to a pesticide cocktail (PEST) at 0.5 μg/L, the maximum allowed in EU drinking water, to assess long-term effects at 28 dpf. Behavioural and molecular changes were also evaluated at 5 dpf, immediately after exposure. By testing a mixture from early development, our approach reflects real-world environmental conditions more accurately than single-compound studies. We found early alterations in stress-response and the HPI axis at 5 dpf. At 28 dpf, fish exposed to PEST during development showed reduced anxiety-like behaviour, similar to juveniles continuously treated with PEST.

### PEST exposure alters stress response and morphology in 5 dpf larvae

Although, as expected, exposure to our stated concentration of PEST did not increase mortality, subtle differences at both behavioural and molecular levels emerged by 5 dpf. Our behavioural results indicate that treatment with PEST did not affect immediate reactions and recovery patterns following a flash of light. However, PEST exposure led to a more pronounced reduction in the total movement of the fish over the repeated acoustic cues. Importantly, this change in habituation was less pronounced in the fish at which the exposure to PEST started 24 h after fertilisation (24hWATER + PEST), when the neuromuscular junction was already formed (Fichi et al. 2019). This difference between treatments could suggest that PEST might be affecting their locomotor activity rather than their stress reactivity, as others have previously shown that pesticides’ exposure might result in either hyperactivity (Hussain et al. 2020) or reduced locomotion (Bridi et al. 2017). Alternatively, our behavioural results could suggest that developmental exposure to PEST interfered with zebrafish’ non-associative learning abilities (García‐González et al. 2021; Beppi et al. 2021).

Nonetheless, our RT-qPCR data confirmed the alterations in stress reactivity since there was a significant reduction in the expression of corticotropin-releasing factor (*crf*) in the PEST-treated fish, thus confirming the alterations in the HPI axis. Similarly, PEST exposure had a significant effect on the expression of genes associated with oxidative stress, which may represent a compensatory mechanism to counteract the neurotoxic effects of PEST. In agreement, previous research has shown that pesticides can increase reactive oxygen species (ROS) levels (Teixeira et al. 2024). However, this response appears to be proportional to the concentration of PEST.

Subtle differences in morphology were also observed. There was an increased head and eye width in the 24hWATER + PEST but not in the fish treated with PEST from 2 h post-fertilisation. Similarly, exposing 3-day old zebrafish to glyphosate alone, at similar dosage as our current research, leads to morphological alterations (Bridi et al. 2017). It is possible that exposure to PEST later in development, once certain structures have already been established, reduces larvae’ adaptive developmental plasticity (Nettle and Bateson 2015), their ability to counteract the impact of these environmental factors. In contrast, early-stage exposure may allow the developing morphology to adapt before its final conformation is reached.

### Early-life PEST exposure leads to reduced anxiety-like behaviours

Our results support the claim that exposing zebrafish to a PEST mixture during the first five days of development had long-lasting effects, as assessed at 28 dpf. This 28 dpf stage was chosen as representing the transition from larval to juvenile phases (Singleman and Holtzman 2014; King et al. 2020).

At the behavioural level, we observed a reduced anxiety-like phenotype in the juvenile fish that were treated with PEST during early development (5dpfPEST + WATER). Importantly, this behavioural change was similarly present in juvenile fish continuously exposed to the same PEST concentration throughout the 28 days (28dpfPEST), suggesting that exposure during a critical developmental window is as detrimental as prolonged exposure. This significant difference in both PEST-treated groups compared to control, alongside the stress-related differences observed at 5 dpf, highlights the potential impact of pesticides on stress-related behaviours. Combined, these findings suggest that pesticide exposure may reduce anxiety-like behaviour by reducing the tone of the HPI axis (Packard et al. 2016).

In this regard, sustained exposure to stressful stimuli during development can lead to adaptive changes that alter responses to environmental stressors later in life, potentially diminishing anxiety-like behaviours in adulthood (Leggieri et al. 2024). In support of this, molecular analysis only revealed a significant dysregulation of the HPI axis in the 28-dpf fish continuously exposed to PEST. Notably, the HPI axis was unaffected in the 5dpfPEST + WATER group. Alternatively, it is possible that what we are seeing in the NTT is not anxiety-like changes mediated via alterations in the functioning of the HPI-axis but rather anhedonia or, more possibly, a diminished awareness of potential threats in a new environment (e.g. attention deficits), as there were no differences in overall movement (Ponzoni et al. 2021; Braida et al. 2023). Finally, neither PEST treatment resulted in morphological or maturational differences at juvenile stages.

## Conclusions and limitations

Our present research indicates that early-life acute exposure to pesticides, even at concentrations deemed as safe, during critical developmental periods can be as harmful as sustained exposure to the same levels. Our study highlights the need for increased caution regarding pesticide exposure, particularly if affecting subjects during developmental stages, as it could affect the overall animal health status.

Aquatic wildlife is simultaneously exposed to a wide array of chemicals. However, the study of chemical mixtures has often been neglected (Kortenkamp et al. 2019). Yet, the combined effects of these chemicals are highly unpredictable, as they may result in synergistic or antagonistic interactions, amongst others (Forner-Piquer et al. 2021b). Therefore, by using a pesticide cocktail, we highlight mixture toxicity, which better reflects real-world exposure.

However, our research does have some limitations. Whilst our data suggest possible HPI axis adaptation to early-life pesticide exposure, we did not assess fertility. As stress adaptations during development often involve allostatic overload and reduced fertility (Eachus et al. 2024; Leggieri et al. 2024), further studies are needed to explore such trade-offs with our pesticide mixture. Similarly, we found no changes in *th1* or *ache* expression, genes linked to pesticide-induced Parkinson’s and Alzheimer’s disease, respectively (Flores-Gutierrez et al. 2023). This may suggest no impact at the gene level, effects emerging later in life, or changes occurring only at the protein level. In addition, variability may also mask effects, as *cyp1a* expression, involved in the metabolism of many exogenous chemicals (Sarasquete and Segner 2000; Ardeshir et al. 2018), approached but did not reach significance in PEST-treated juveniles.

## Supplementary Information

Below is the link to the electronic supplementary material.

Supplementary Material 1.

## Data Availability

Coding generated for this manuscript and additional information are available within Supplementary Material [see Supplementary Information (SI)]. Raw data can be kindly requested to corresponding authors.
