# Supplementary material for: Zebrafish exposed to a cocktail of pesticides during early development display long-lasting neurobehavioral alterations
Source: Arch Toxicol. 2025 Aug 25;99(10):4181–95. doi: 10.1007/s00204-025-04129-6 (PMC12454604; doi:10.1007/s00204-025-04129-6)
Supplement: Supplementary file 1 — Supplementary Material 1. [file 204_2025_4129_MOESM1_ESM.docx]

**Sup. Table 1**. Genes and corresponding primers used for qPCR experiments.

| Type / Purpose | Name | ACCESSION NUMBER | Sequence (5’ to 3’)  Forward  Reverse | Product size (bp) | Efficiency (%) | References |
| --- | --- | --- | --- | --- | --- | --- |
| Housekeeping | actin, beta 2 (*actb2*) | NM_181601.5 | CGAGCTGTCTTCCCATCCA | 86 | 95 | <https://doi.org/10.3389/fnbeh.2015.00271> |
|  |  |  | TCACCAACGTAGCTGTCTTTCTG |  |  |  |
|  | ribosomal protein L13a (*rpl13a*) | NM_212784.1 | TCTGGAGGACTGTAAGAGGTATGC | 148 | 80 | <https://doi.org/10.3389/fnbeh.2015.00271> |
|  |  |  | AGACGCACAATCTTGAGAGCAG |  |  |  |
| Hypothalamic-pituitary-interrenal (HPI) axis | corticotropin-releasing factor (*crf*) | NM_001007379.1 | CGAGACATCCCAGTATCCAAAAAG | 60 | 97.5 | https://doi.org/10.1371/journal.pone.0175420.t001 |
|  |  |  | TCCAACAGACGCTGCGTTAA |  |  |  |
|  | mineralocorticoid receptor (*mr*) | NM_001100403 | CTTCCAGGTTTCCGCAGTCTAC | 75 | 97.7 | https://doi.org/10.1371/journal.pone.0175420.t003 |
|  |  |  | GGAGGAGAGACACATCCAGGAAT |  |  |  |
|  | glucocorticoid receptor α (*grα*) | NM_001020711.3 | ACTCCATGCACGACTTGGTG | 90 | 86.11 | https://doi.org/10.1371/journal.pone.0175420.t004 |
|  |  |  | GCATTTCGGGAAACTCCACG |  |  |  |
| Oxidative stress | catalase (*cat*) | NM_130912.2 | TGAGGCTGGGTCATCAGATA | 138 | 96.6 | <https://doi.org/10.1016/j.etap.2016.03.010> |
|  |  |  | AAAGACGGAAACAGAAGCGT |  |  |  |
|  | superoxide dismutase 1 (*sod1* (*esod1*)) | NM_131294.1 | CGTCTATTTCAATCAAGAGGGTG | 124 | 89.2 | <https://doi.org/10.1016/j.chemosphere.2018.09.105> |
|  |  |  | GATGCAGCCGTTTGTGTTGTC |  |  |  |
|  | superoxide dismutase 2 (*sod2* (*fsod2*)) | NM_199976.1 | CTTGGGATAGATGTCTGGG | 216 | 87.7 | <https://doi.org/10.1016/j.chemosphere.2018.09.105> |
|  |  |  | GTGGTCTGATTAATTGTGCG |  |  |  |
| Pesticide metabolism | tyrosine hydroxylase 1 (*th1* (*cth1*)) | NM_131149.1 | GACGGAAGATGATCGGAGACA | 95 | 83.1 | <https://doi.org/10.1016/j.chemosphere.2018.09.105> |
|  |  |  | CCGCCATGTTCCGATTTCT |  |  |  |
|  | cyto-chrome P4501A family 1 subfamily A (*cyp1a*) | NM_131879.2 | AGGACAACATCAGAGACATCACCG | 179 | 88.6 | [https://doi.org/10.1002/tox.22035. Epub 2014 Aug 6](https://doi.org/10.1002/tox.22035.%20Epub%202014%20Aug%206) |
|  |  |  | CACTAGATAGACAACCGCCCAGG |  |  |  |
|  | Acetylcholinesterase (*ache*) | NM_131846.2 | CATACGCACAATACGCTGCC | 118 | 98.1 | <https://doi.org/10.1016/j.etap.2016.03.010> |
|  |  |  | TACACAGCACCATGCGAGTT |  |  |  |

**Sup. Table 2**. Visually scoring maturation of fish based on pigment of the skin, tail fin, anal fin and dorsal fin.

| **Group** | **Pigment Average** | **Tail Average** | **Anal Average** | **Dorsal Average** | **Total Average** |
| --- | --- | --- | --- | --- | --- |
| **28dpfH_2_O-A** | 2 | 2 | 2 | 2 | 2 |
| **28dpfH_2_O-B** | 2 | 2 | 3 | 3 | 3 |
| **5dpfPEST-A** | 2 | 3 | 3 | 3 | 3 |
| **5dpfPEST-B** | 2 | 2 | 3 | 3 | 3 |
| **28dpfPEST-A** | 2 | 2 | 2 | 2 | 2 |
| **28dpfPEST-B** | 3 | 3 | 3 | 3 | 3 |

The average rating for each aspect was taken and rounded to the nearest whole number. The overall rating is seen in the Total Average Column. No statistical differences where observed. A and B indicate replicates for each treatment (n = 8). Scoring system following Singleman & Holtzman, 2014 (doi: 10.1089/zeb.2014.0976).


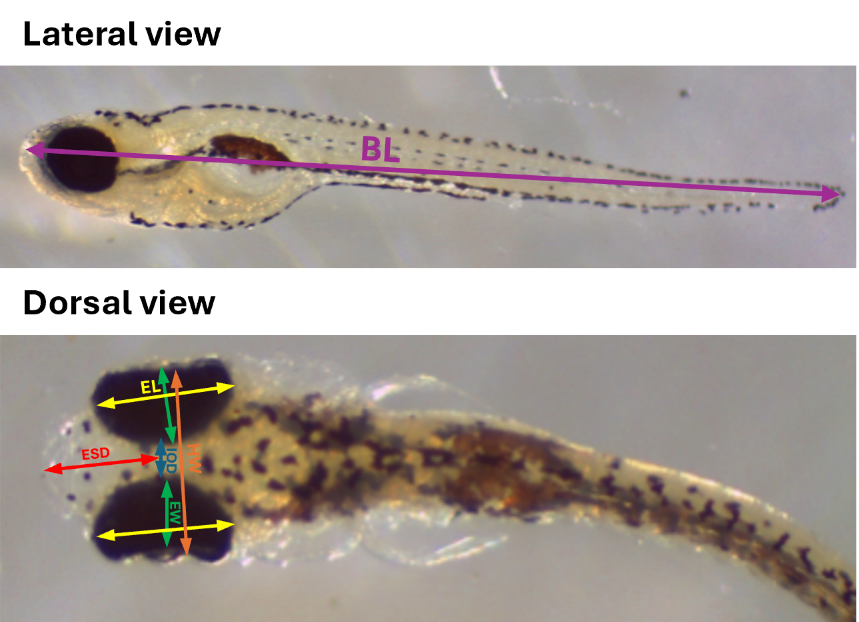


**Sup. Fig. 1. Zebrafish measurements taken at 5 dpf from intact fixed larvae.** Representative images for a zebrafish larva. From the lateral-view (above image), we took Body Length (BL, purple), while the dorsal-view (below) images was used to calculate Eye-Snout Distance (ESD, red), Head Width (HW, orange), Inter-Ocular Distance (IOD, blue), Eye Width (EW; both left and right; green), and Eye Length (EL; both left and right; yellow). Pictures of all animals were taken at similar magnification.


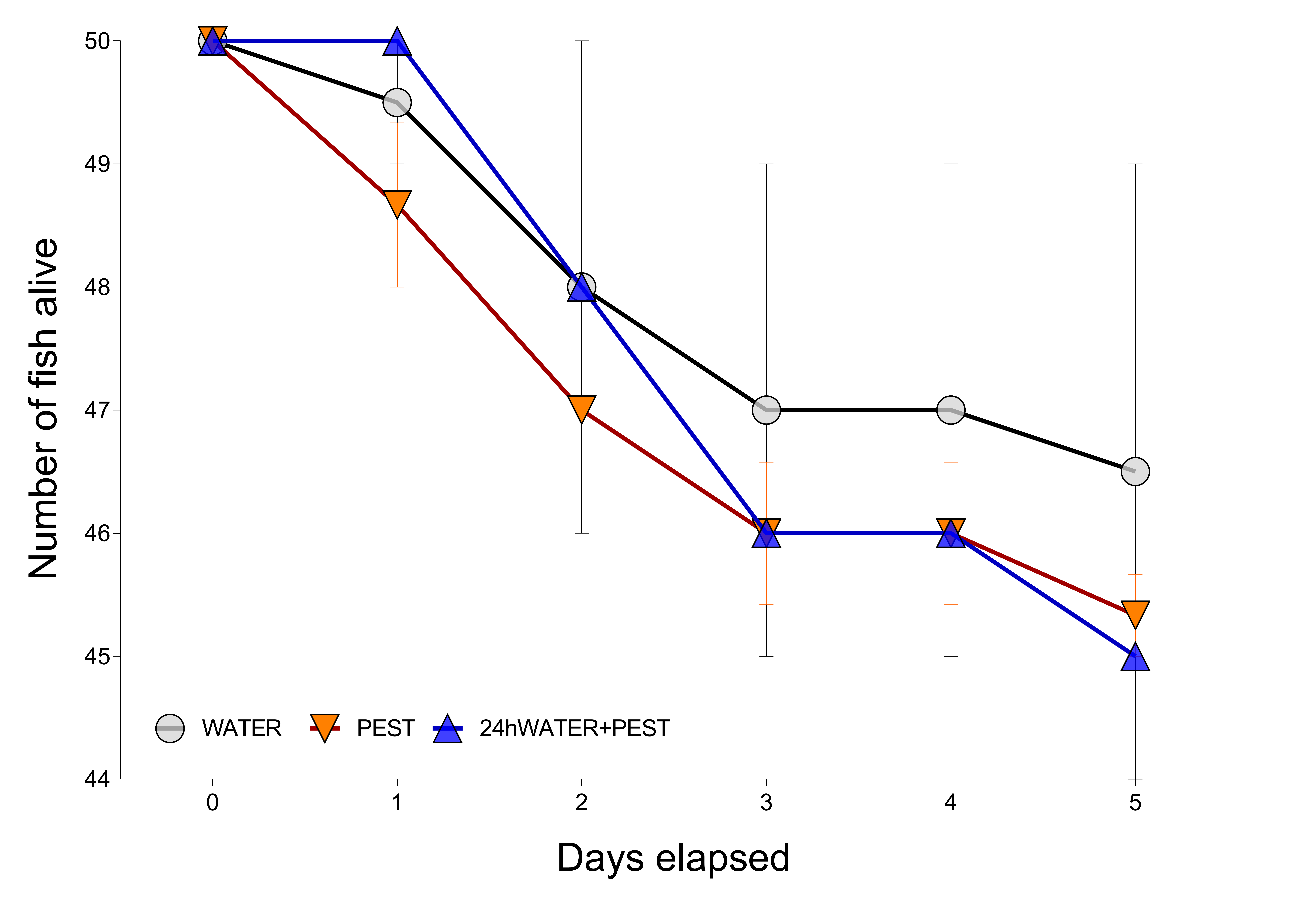


**Sup. Fig. 2**. Assessing survival rate of zebrafish embryos during the first 5 dpf. Eggs were collected and pulled together for experimentation at around 2 hours post-fertilization. Then, eggs were organised in groups of 50 per petri dish and subjected to corresponding treatment group. Each day, number of eggs/embryos that were still alive were quantified. The dead individuals were removed to prevent contamination. Graph shows mean number of subjects alive for each treatment ± Standard Error Meam (SEM). n = 2-3 per group with 50 individuals as starting point in each. Treatments: WATER: Negative control with just water, PEST: Eggs exposed to PEST from around 2 hpf to 5 dpf, and 24hWATER+ PEST: Exposed to water for the initial 24 hpf followed by PEST for the remaining 4 days. The mixed-effect model did not reveal any effect of the factor ‘treatment’ (F (0.9938, 1.988) = 0.3624, p = 0.4969) nor the factor ‘time’ (F (1.200, 2.400) = 10.23, p = 0.24). Similarly, the interaction ‘time x treatment’ was not significant (F (1.660, 0.3319) = 0.3258, p = 0.1660).


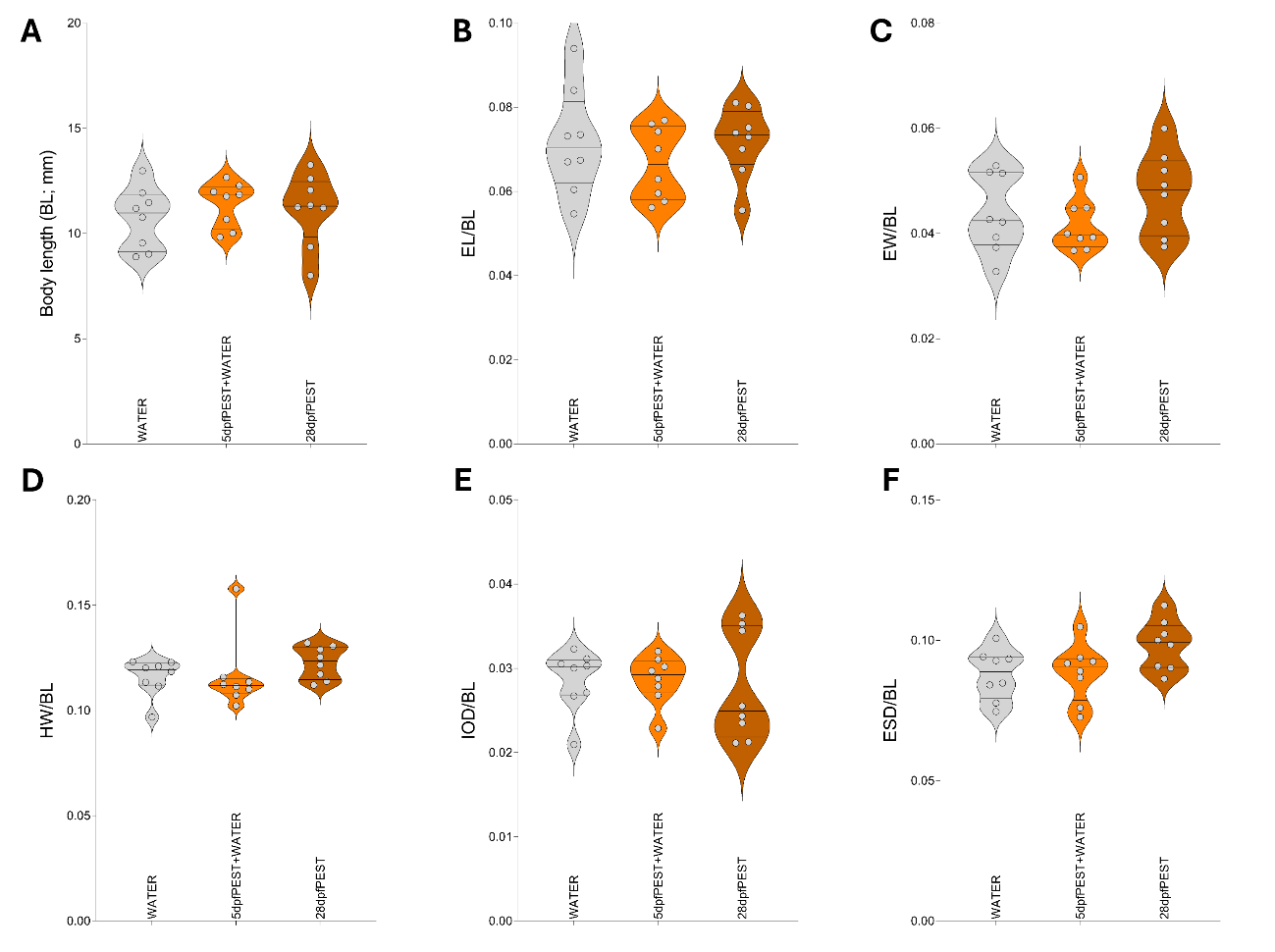


**Sup. Fig. 3. Morphological assessment of 28 dpf fish from Experiment 2.** Morphological assessment of fixed larvae following behavioural assessment, including following measures: **A)** total body length (BL), **B)** eye length (EL), **C)** eye width (EW), **D)** head width (HW), **E)** inter-ocular distance (IOD), **F)** eye-snout distance (ESD). All measures corrected by BL, except BL itself. Violin plots include points as single values

**# Code for experiment 1**

# Load required libraries

library(lme4)

library(lmerTest)

library(emmeans)

library(car)

library(ggplot2)

library(dplyr)

library(tidyr)

library(betareg)

library(MASS)

library(nortest) # For Anderson-Darling and Kolmogorov-Smirnov tests

# Load and preprocess data

# Step 1: Add fish_ID as a unique identifier

num_fish <- nrow(data_clean) / 718 # Calculate the number of fish based on 718 rows per fish

fish_ID_vector <- rep(1:num_fish, each = 718) # Repeat each fish ID 718 times

data_clean <- data_clean %>%

mutate(fish_ID = fish_ID_vector)

# Step 2: Add 'second' as a new variable (0-717 for each fish)

second <- 0:717

data_clean <- data_clean %>%

mutate(second = rep(second, times = num_fish))

# Step 3: Convert relevant columns to factors

data_clean$Treatment <- as.factor(data_clean$Treatment)

data_clean$fish_ID <- as.factor(data_clean$fish_ID)

# Ensure Distance_moved_total is numeric

data_clean$Distance_moved_total <- as.numeric(data_clean$Distance_moved_total)

# Check for NA values in Distance_moved_total

cat("Number of NA values in Distance_moved_total:", sum(is.na(data_clean$Distance_moved_total)), "\n")

## 1. Basal Activity Analysis (0–60s)

# Subset data for basal activity periods

basal_data_1 <- data_clean %>%

filter(second >= 1 & second <= 60)

# Linear model to detect outliers in Basal 1

outlier_result_basal_1 <- outlierTest(lm(Distance_moved_total ~ Treatment * second, data = basal_data_1))

# Extract and remove outliers if detected

if (!is.null(outlier_result_basal_1)) {

outliers_basal_1 <- as.numeric(names(outlier_result_basal_1$rstudent))

cat(length(outliers_basal_1), "outliers removed from Basal 1.\n")

basal_data_1_clean <- basal_data_1[-outliers_basal_1, ]

} else {

cat("No outliers detected in Basal 1.\n")

basal_data_1_clean <- basal_data_1

}

# Normality tests (Kolmogorov-Smirnov and Anderson-Darling)

ks_test_result_basal_1_without_outliers <- ks.test(basal_data_1_clean$Distance_moved_total, "pnorm",

mean = mean(basal_data_1_clean$Distance_moved_total),

sd = sd(basal_data_1_clean$Distance_moved_total))

print(ks_test_result_basal_1_without_outliers)

ad_test_result_basal_1_without_outliers <- ad.test(basal_data_1_clean$Distance_moved_total)

print(ad_test_result_basal_1_without_outliers)

# Homogeneity of variances test (Levene's test)

levene_test_basal_1_without_outliers <- leveneTest(Distance_moved_total ~ Treatment, data = basal_data_1_clean)

print(levene_test_basal_1_without_outliers)

# Adjust non-positive values proportionally

if (any(basal_data_1_clean$Distance_moved_total <= 0)) {

min_value <- abs(min(basal_data_1_clean$Distance_moved_total, na.rm = TRUE))

basal_data_1_clean$Distance_moved_total <- basal_data_1_clean$Distance_moved_total + min_value + 0.00001

}

# Fit Gamma distribution and plot histogram

library(MASS)

gamma_fit_clean <- fitdistr(basal_data_1_clean$Distance_moved_total, "gamma")

hist(basal_data_1_clean$Distance_moved_total, freq = FALSE, breaks = 30,

main = "Histogram with Gamma Fit (Without Outliers)")

curve(dgamma(x, shape = gamma_fit_clean$estimate[1], rate = gamma_fit_clean$estimate[2]),

col = "red", lwd = 2, add = TRUE)

# Generalized Linear Mixed Model (GLMM) with Gamma distribution

# Fit GLMM with Gamma distribution

glmm_model_clean <- glmer(Distance_moved_total ~ Treatment + (1 | fish_ID),

family = Gamma(link = "log"), data = basal_data_1_clean)

summary(glmm_model_clean)

# Likelihood ratio test

anova(glmer(Distance_moved_total ~ (1 | fish_ID), family = Gamma(link = "log"), data = basal_data_1_clean),

glmm_model_clean, test = "Chisq")

# Set H2O as the reference group

basal_data_1_clean$Treatment <- relevel(basal_data_1_clean$Treatment, ref = "H2O")

summary(glmer(Distance_moved_total ~ Treatment + (1 | fish_ID),

family = Gamma(link = "log"), data = basal_data_1_clean))

## 2. Basal 2 Activity Analysis (122–362s)

# Subset data for Basal 2 period (4 min after flash)

basal_data_2 <- data_clean %>%

filter(second >= 122 & second <= 362)

# Linear model to detect outliers in Basal 2

outlier_result_basal_2 <- outlierTest(lm(Distance_moved_total ~ Treatment * second, data = basal_data_2))

# Extract and remove outliers if detected

if (!is.null(outlier_result_basal_2)) {

outliers_basal_2 <- as.numeric(names(outlier_result_basal_2$rstudent))

cat(length(outliers_basal_2), "outliers removed from Basal 2.\n")

basal_data_2_clean <- basal_data_2[-outliers_basal_2, ]

} else {

cat("No outliers detected in Basal 2.\n")

basal_data_2_clean <- basal_data_2

}

# Normality tests (Kolmogorov-Smirnov and Anderson-Darling)

ks_test_result_basal_2_without_outliers <- ks.test(basal_data_2_clean$Distance_moved_total, "pnorm",

mean = mean(basal_data_2_clean$Distance_moved_total),

sd = sd(basal_data_2_clean$Distance_moved_total))

print(ks_test_result_basal_2_without_outliers)

ad_test_result_basal_2_without_outliers <- ad.test(basal_data_2_clean$Distance_moved_total)

print(ad_test_result_basal_2_without_outliers)

# Homogeneity of variances test (Levene's test)

levene_test_basal_2_without_outliers <- leveneTest(Distance_moved_total ~ Treatment, data = basal_data_2_clean)

print(levene_test_basal_2_without_outliers)

# GLMM for Basal 2 period

glmm_model_h2o_ref2 <- glmer(Distance_moved_total ~ Treatment + (1 | fish_ID),

family = Gamma(link = "log"),

data = basal_data_2_clean)

summary(glmm_model_h2o_ref2)

# Likelihood ratio test

anova(glmer(Distance_moved_total ~ (1 | fish_ID), family = Gamma(link = "log"), data = basal_data_2_clean),

glmm_model_h2o_ref2, test = "Chisq")

## 3. Recovery Rate to First Flash of Light (60s–81s)

# Subset the data for 20 seconds after the flash (62s to 81s)

post_flash_20s <- subset(data_clean, second >= 62 & second <= 81)

# Identify outliers in Distance_moved_total before model fitting

library(car)

outlier_result_20s <- outlierTest(lm(Distance_moved_total ~ Treatment * second, data = post_flash_20s))

# Extract and remove outliers if detected

if (!is.null(outlier_result_20s)) {

outliers_20s <- as.numeric(names(outlier_result_20s$rstudent))

print(outliers_20s)

post_flash_20s_clean <- post_flash_20s[-outliers_20s, ]

cat(length(outliers_20s), "outliers removed.\n")

} else {

post_flash_20s_clean <- post_flash_20s

cat("No outliers detected.\n")

}

# Normality tests (without outliers)

ks_test_clean <- ks.test(post_flash_20s_clean$Distance_moved_total, "pnorm",

mean = mean(post_flash_20s_clean$Distance_moved_total, na.rm = TRUE),

sd = sd(post_flash_20s_clean$Distance_moved_total, na.rm = TRUE))

print(ks_test_clean)

ad_test_clean <- ad.test(post_flash_20s_clean$Distance_moved_total)

print(ad_test_clean)

# Homogeneity of variances test (Levene's test)

levene_clean <- leveneTest(Distance_moved_total ~ Treatment, data = post_flash_20s_clean)

print(levene_clean)

# Ensure all values are positive (proportional shift)

if (min(post_flash_20s$Distance_moved_total, na.rm = TRUE) <= 0) {

scale_factor <- abs(min(post_flash_20s$Distance_moved_total, na.rm = TRUE)) + 0.00001

post_flash_20s$Distance_moved_total <- post_flash_20s$Distance_moved_total + scale_factor

}

if (min(post_flash_20s_clean$Distance_moved_total, na.rm = TRUE) <= 0) {

scale_factor_clean <- abs(min(post_flash_20s_clean$Distance_moved_total, na.rm = TRUE)) + 0.00001

post_flash_20s_clean$Distance_moved_total <- post_flash_20s_clean$Distance_moved_total + scale_factor_clean

}

# Review adjusted values

summary(post_flash_20s$Distance_moved_total)

summary(post_flash_20s_clean$Distance_moved_total)

# Export filtered data to CSV

write.csv(post_flash_20s_clean, "filtered_data_clean_20s.csv", row.names = FALSE)

# Fit glmmTMB models (Gamma, log link)

# a) With Outliers

glmmTMB_20s_with_outliers <- glmmTMB(

Distance_moved_total ~ Treatment * second + (1 | fish_ID),

data = post_flash_20s,

family = Gamma(link = "log")

)

summary(glmmTMB_20s_with_outliers)

# b) Without Outliers

post_flash_20s$Treatment <- relevel(post_flash_20s$Treatment, ref = "24hPEST")

glmmTMB_20s_without_outliers <- glmmTMB(

Distance_moved_total ~ Treatment * second + (1 | fish_ID),

data = post_flash_20s_clean,

family = Gamma(link = "log")

)

summary(glmmTMB_20s_without_outliers)

# Post-hoc Analysis

emmeans_20s_with_outliers <- emmeans(glmmTMB_20s_with_outliers, pairwise ~ Treatment, type = "response")

summary(emmeans_20s_with_outliers)

emmeans_20s_without_outliers <- emmeans(glmmTMB_20s_without_outliers, pairwise ~ Treatment, type = "response")

summary(emmeans_20s_without_outliers)

# Change reference group to H2O

post_flash_20s_clean$Treatment <- relevel(post_flash_20s_clean$Treatment, ref = "H2O")

glmmTMB_h2o_ref <- glmmTMB(

Distance_moved_total ~ Treatment * second + (1 | fish_ID),

data = post_flash_20s_clean,

family = Gamma(link = "log")

)

summary(glmmTMB_h2o_ref)

# Post-hoc comparisons

posthoc_h2o_ref <- emmeans(glmmTMB_h2o_ref, pairwise ~ Treatment | second, type = "response")

summary(posthoc_h2o_ref$emmeans)

summary(posthoc_h2o_ref$contrasts)

## 4. Amount of jump in response to the light

# Check for the presence of seconds 59, 60, and 61 in the original dataset (Total Distance)

seconds_check <- data_clean %>%

filter(second %in% c(59, 60, 61)) %>%

group_by(fish_ID) %>%

summarise(count_59 = sum(second == 59),

count_60 = sum(second == 60),

count_61 = sum(second == 61),

.groups = 'drop')

print(seconds_check)

# Subset the data for seconds 59 to 61 (Total Distance)

data_clean.lightjump <- subset(data_clean, second %in% c(59, 60, 61))

# Calculate the change in activity before and after the flash (Total Distance)

jump_data_total_distance <- data_clean.lightjump %>%

group_by(fish_ID, Treatment) %>%

summarize(

mean_before_flash_total = mean(Distance_moved_total[second == 59], na.rm = TRUE), # Movement before flash (second 59)

mean_during_flash_total = mean(Distance_moved_total[second == 60], na.rm = TRUE), # Movement during flash (second 60)

mean_after_flash_total = mean(Distance_moved_total[second == 61], na.rm = TRUE), # Movement after flash (second 61)

jump_total_distance = mean_after_flash_total - mean_before_flash_total, # Jump based on difference in total distance

.groups = 'drop'

)

# Check the resulting jump_data (Total Distance)

print(jump_data_total_distance)

# Ensure 'Treatment' is a factor (Total Distance)

jump_data_total_distance$Treatment <- as.factor(jump_data_total_distance$Treatment)

# Check the levels of 'Treatment' (Total Distance)

print(levels(jump_data_total_distance$Treatment))

# Check for NA values (Total Distance)

print(anyNA(jump_data_total_distance))

# Identify and remove outliers

cat("\n### Identifying outliers\n")

outlier_result <- outlierTest(lm(jump_total_distance ~ Treatment, data = jump_data_total_distance))

if (!is.null(outlier_result)) {

outliers <- as.numeric(names(outlier_result$rstudent))

cat(length(outliers), "outliers detected.\n")

# Create datasets with and without outliers

jump_data_with_outliers <- jump_data_total_distance

jump_data_clean <- jump_data_total_distance[-outliers, ]

} else {

jump_data_with_outliers <- jump_data_total_distance

jump_data_clean <- jump_data_total_distance

cat("No outliers detected.\n")

}

print(outlier_result)

# Normality and Levene's test (without outliers)

cat("\n### Normality test (without outliers)\n")

ks_test_clean <- ks.test(

jump_data_clean$jump_total_distance,

"pnorm",

mean = mean(jump_data_clean$jump_total_distance, na.rm = TRUE),

sd = sd(jump_data_clean$jump_total_distance, na.rm = TRUE)

)

print(ks_test_clean)

ad_test_clean <- ad.test(jump_data_clean$jump_total_distance)

print(ad_test_clean)

cat("\n### Levene's test (without outliers)\n")

levene_test_clean <- leveneTest(jump_total_distance ~ Treatment, data = jump_data_clean)

print(levene_test_clean)

# Ensure non-positive values are adjusted (GLMM only)

cat("\n### Adjusting for non-positive values (GLMM only)\n")

if (any(jump_data_with_outliers$jump_total_distance <= 0)) {

min_value <- abs(min(jump_data_with_outliers$jump_total_distance, na.rm = TRUE))

jump_data_with_outliers$jump_total_distance <- jump_data_with_outliers$jump_total_distance + min_value + 0.00001

cat("Adjusted non-positive values in jump_total_distance for GLMM.\n")

}

# Verify adjustment

if (any(jump_data_with_outliers$jump_total_distance <= 0)) {

stop("Error: jump_total_distance still contains non-positive values after adjustment.")

}

# GLMM with Gamma distribution (log-link) with interaction (Treatment × Time)

model_recovery <- glmer(distance_moved ~ treatment * time + (1|fish_id), data=data, family=Gamma(link="log"))

summary(model_recovery)

# Post-hoc comparisons

emmeans(model_recovery, pairwise ~ treatment * time, adjust="bonferroni")

## 5. Burst activity response to flash (60s–62s)

# Linear model (LM) to analyze burst activity after the first flash

cat("\n### Fitting LM (without outliers)\n")

lm_without_outliers <- lm(jump_total_distance ~ Treatment, data = jump_data_clean)

summary(lm_without_outliers)

# Post-hoc pairwise comparisons (LM)

emmeans_lm_clean <- emmeans(lm_without_outliers, pairwise ~ Treatment)

cat("\n### Post-hoc results (LM without outliers)\n")

summary(emmeans_lm_clean)

# Change reference group to H2O

jump_data_clean$Treatment <- relevel(jump_data_clean$Treatment, ref = "H2O")

# Fit linear model (LM) without outliers

lm_h2o_ref <- lm(jump_total_distance ~ Treatment, data = jump_data_clean)

summary(lm_h2o_ref)

# Post-hoc comparisons (EMMeans)

emmeans_lm_h2o <- emmeans(lm_h2o_ref, pairwise ~ Treatment)

summary(emmeans_lm_h2o)

## 5. Startle response habituation (62s–662s)

# GLMM (Binomial distribution) to evaluate habituation to acoustic stimuli

model_startle <- glmer(response ~ treatment * tap_number + (1|fish_id), data=data, family=binomial)

summary(model_startle)

# Post-hoc comparisons

emmeans(model_startle, pairwise ~ treatment * tap_number, adjust="bonferroni")

## 6. Assigning tap numbers and response classification

# Calculate mean and standard deviation during the basal period

mean_basal <- mean(basal_data_1$Distance_moved_total)

sd_basal <- sd(basal_data_1$Distance_moved_total)

# Set the response threshold as mean + 1 * SD

threshold <- mean_basal + 1 * sd_basal

# Create an empty dataframe to store all tap events

tap_all_25 <- data.frame()

# Generate the 25 tap events using a loop

for (i in 1:25) {

# Calculate the seconds corresponding to each tap event

start_time <- 659 + (i - 1) * 2

end_time <- start_time + 1

# Subset for each tap event

tap_event <- subset(data_clean, second == start_time | second == end_time)

# Add the column with the tap number

tap_event$tap.number <- i

# Adjust time relative to tap onset

tap_event$time.adj <- tap_event$second - start_time

# Combine tap events into the final dataframe

tap_all_25 <- rbind(tap_all_25, tap_event)

}

# Create a binary 'response' variable based on the threshold

tap_all_25$response <- ifelse(tap_all_25$Distance_moved_total > threshold, 1, 0)

# Calculate the percentage of responders for each tap and treatment group

tap_responses <- aggregate(response ~ tap.number + Treatment, data = tap_all_25, FUN = mean)

tap_responses$percentage_responders <- tap_responses$response * 100 # Convert to percentage

# Save the tap_responses table for later use

write.csv(tap_responses, "tap_responses_percentage3.csv", row.names = FALSE)

# Print to verify the result

print(tap_responses)

# Load the betareg package

library(betareg)

# Ensure that the percentage of responders is within the (0, 1) interval

tap_responses$percentage_responders <- tap_responses$percentage_responders / 100

tap_responses$percentage_responders <- ifelse(tap_responses$percentage_responders == 1, 0.999, tap_responses$percentage_responders)

tap_responses$percentage_responders <- ifelse(tap_responses$percentage_responders == 0, 0.001, tap_responses$percentage_responders)

# Fit the beta regression model with all taps included

beta_model <- betareg(percentage_responders ~ Treatment * tap.number, data = tap_responses)

# Summary of the beta regression model

summary(beta_model)

# Identify movement for the first and last tap

tap_all_25 <- tap_all_25 %>%

mutate(

Moved_First_Tap = ifelse(tap.number == 1 & Distance_moved_total > threshold, TRUE, FALSE),

Moved_Last_Tap = ifelse(tap.number == 25 & Distance_moved_total > threshold, TRUE, FALSE)

)

# Summarize movement for each fish across treatments

moved_summary <- tap_all_25 %>%

group_by(Treatment, fish_ID) %>%

summarise(

Moved_First_Tap = any(Moved_First_Tap),

Moved_Last_Tap = any(Moved_Last_Tap)

)

# Calculate the percentage of fish that moved at the first and last tap per treatment

moved_percent <- moved_summary %>%

group_by(Treatment) %>%

summarise(

Percent_Moved_First = mean(Moved_First_Tap) * 100,

Percent_Moved_Last = mean(Moved_Last_Tap) * 100

)

# Print the result to verify

print(moved_percent)

# Fit the logistic regression model for the first tap

logit_first_tap <- glm(Moved_First_Tap ~ Treatment, data = moved_summary, family = "binomial")

# Perform post-hoc pairwise comparisons for the first tap

pairwise_first_tap <- emmeans(logit_first_tap, pairwise ~ Treatment, adjust = "tukey")

# Fit the logistic regression model for the last tap

logit_last_tap <- glm(Moved_Last_Tap ~ Treatment, data = moved_summary, family = "binomial")

# Perform post-hoc pairwise comparisons for the last tap

pairwise_last_tap <- emmeans(logit_last_tap, pairwise ~ Treatment, adjust = "tukey")

# Print the pairwise comparison results

print(pairwise_first_tap)

print(pairwise_last_tap)

unique(tap_responses$tap.number)

# Perform post-hoc pairwise comparisons for all tap numbers

emmeans_results_all_taps <- emmeans(beta_model, pairwise ~ Treatment | tap.number, adjust = "tukey", at = list(tap.number = unique(tap_responses$tap.number)))

# Print the results for all taps

summary(emmeans_results_all_taps)

## 7. Total movement per tap (662s–710s)

# Create an empty dataframe to store all tap events

tap_all_25 <- data.frame()

# Generate 25 tap events using a loop

for (i in 1:25) {

# Calculate the seconds corresponding to each tap event

start_time <- 659 + (i - 1) * 2

end_time <- start_time + 1

# Subset the data for each tap event

tap_event <- subset(data_clean, second == start_time | second == end_time)

# Add a column with the tap number

tap_event$tap.number <- i

# Adjust the time relative to the start of the tap

tap_event$time.adj <- tap_event$second - start_time

# Combine the tap events into the final dataframe

tap_all_25 <- rbind(tap_all_25, tap_event)

}

# Calculate slopes or differences in total movement by tap number and treatment

tap_slopes_25 <- tap_all_25 %>%

group_by(tap.number, fish_ID, Treatment) %>%

summarise(Distance_moved_total = mean(Distance_moved_total, na.rm = TRUE), .groups = "drop")

# Ensure Treatment and tap number variables are correctly categorized

tap_slopes_25$Treatment <- as.factor(tap_slopes_25$Treatment)

tap_slopes_25$tap.number <- as.integer(tap_slopes_25$tap.number)

# Identify outliers and create tap_slopes_clean

outlier_result <- outlierTest(

lm(Distance_moved_total ~ Treatment * tap.number, data = tap_slopes_25)

)

if (!is.null(outlier_result)) {

outliers <- as.numeric(names(outlier_result$rstudent))

cat("Outliers detected at row indices:", outliers, "\n")

tap_slopes_clean <- tap_slopes_25[-outliers, ]

cat("tap_slopes_clean created. Number of rows removed:", length(outliers), "\n")

} else {

cat("No outliers detected.\n")

tap_slopes_clean <- tap_slopes_25

}

# Normality & Levene's test without outliers

ks_test_clean <- ks.test(

tap_slopes_clean$Distance_moved_total,

"pnorm",

mean = mean(tap_slopes_clean$Distance_moved_total, na.rm = TRUE),

sd = sd(tap_slopes_clean$Distance_moved_total, na.rm = TRUE)

)

ad_test_clean <- ad.test(tap_slopes_clean$Distance_moved_total)

levene_test_clean <- leveneTest(Distance_moved_total ~ Treatment, data = tap_slopes_clean)

# Check for non-positive values

if (any(tap_slopes_25$Distance_moved_total <= 0)) {

min_value <- abs(min(tap_slopes_25$Distance_moved_total, na.rm = TRUE))

tap_slopes_25$Distance_moved_total <- tap_slopes_25$Distance_moved_total + min_value + 0.00001

}

# Fit GLMM models

cat("\n### Fitting glmmTMB with Outliers (Gamma)\n")

glmmTMB_with_outliers <- glmmTMB(

Distance_moved_total ~ Treatment * tap.number + (1 | fish_ID),

family = Gamma(link = "log"),

data = tap_slopes_25

)

summary(glmmTMB_with_outliers)

cat("\n### Fitting glmmTMB without Outliers (Gamma)\n")

glmmTMB_without_outliers <- glmmTMB(

Distance_moved_total ~ Treatment * tap.number + (1 | fish_ID),

family = Gamma(link = "log"),

data = tap_slopes_clean

)

summary(glmmTMB_without_outliers)

# Post-hoc pairwise comparisons

emmeans_without_outliers <- emmeans(

glmmTMB_without_outliers,

pairwise ~ Treatment | tap.number,

type = "response"

)

summary(emmeans_without_outliers)

# Set H2O as the reference category

tap_slopes_25$Treatment <- relevel(tap_slopes_25$Treatment, ref = "H2O")

tap_slopes_clean$Treatment <- relevel(tap_slopes_clean$Treatment, ref = "H2O")

# GLMM without outliers (Gamma, log link)

glmmTMB_without_outliers <- glmmTMB(

Distance_moved_total ~ Treatment * tap.number + (1 | fish_ID),

family = Gamma(link = "log"),

data = tap_slopes_clean

)

summary(glmmTMB_without_outliers)

# Post-hoc analysis

taps_original <- 1:25

emmeans_without_outliers <- emmeans(

glmmTMB_without_outliers,

pairwise ~ Treatment | tap.number,

at = list(tap.number = taps_original),

type = "response"

)

summary(emmeans_without_outliers)

# GLMM with Gamma distribution (log-link) to assess habituation over repeated taps

model_tap_movement <- glmer(distance_moved ~ treatment * tap_number + (1|fish_id), data=data, family=Gamma(link="log"))

summary(model_tap_movement)

# Post-hoc comparisons

emmeans(model_tap_movement, pairwise ~ treatment * tap_number, adjust="bonferroni")

## 8. Rate of recovery to the second flash of light

## A) Amount of jump in response to the second light

# Check for the presence of seconds 714, 715, and 716 in the original dataset (Total Distance for Second Flash)

seconds_check_second_flash <- data_clean %>%

filter(second %in% c(714, 715, 716)) %>%

group_by(fish_ID) %>%

summarise(count_714 = sum(second == 714),

count_715 = sum(second == 715),

count_716 = sum(second == 716),

.groups = 'drop')

print(seconds_check_second_flash)

# Subset the data for seconds 714 to 716 (Total Distance for Second Flash)

data_clean.second_flash <- subset(data_clean, second %in% c(714, 715, 716))

# Calculate the change in activity before and after the second flash (Total Distance)

jump_data_total_distance_second_flash <- data_clean.second_flash %>%

group_by(fish_ID, Treatment) %>%

summarize(

mean_before_flash_total_second = mean(Distance_moved_total[second == 714], na.rm = TRUE), # Movement before second flash

mean_during_flash_total_second = mean(Distance_moved_total[second == 715], na.rm = TRUE), # Movement during second flash

mean_after_flash_total_second = mean(Distance_moved_total[second == 716], na.rm = TRUE), # Movement after second flash

jump_total_distance_second_flash = mean_after_flash_total_second - mean_before_flash_total_second, # Jump based on difference in total distance

.groups = 'drop'

)

# Check the resulting jump_data (Total Distance for Second Flash)

print(jump_data_total_distance_second_flash)

# Ensure 'Treatment' is a factor (Total Distance for Second Flash)

jump_data_total_distance_second_flash$Treatment <- as.factor(jump_data_total_distance_second_flash$Treatment)

# Check the levels of 'Treatment' (Total Distance for Second Flash)

print(levels(jump_data_total_distance_second_flash$Treatment))

# Check for NA values (Total Distance for Second Flash)

print(anyNA(jump_data_total_distance_second_flash))

# Check and remove outliers (Total Distance for Second Flash)

outlier_test <- outlierTest(lm(jump_total_distance_second_flash ~ Treatment, data = jump_data_total_distance_second_flash))

if (!is.null(outlier_test)) {

outliers <- as.numeric(names(outlier_test$rstudent))

jump_data_total_distance_second_flash_clean <- jump_data_total_distance_second_flash[-outliers, ]

cat(length(outliers), "outliers removed.\n")

} else {

jump_data_total_distance_second_flash_clean <- jump_data_total_distance_second_flash

cat("No outliers detected.\n")

}

# Normality tests (Without Outliers)

cat("\n### Normality tests (Without Outliers)\n")

shapiro_without_outliers <- shapiro.test(jump_data_total_distance_second_flash_clean$jump_total_distance_second_flash)

ad_without_outliers <- ad.test(jump_data_total_distance_second_flash_clean$jump_total_distance_second_flash)

print(shapiro_without_outliers)

print(ad_without_outliers)

# Levene's Test for Homogeneity of Variances (Without Outliers)

levene_without_outliers <- leveneTest(jump_total_distance_second_flash ~ Treatment, data = jump_data_total_distance_second_flash_clean)

cat("\n### Levene's test (Without Outliers)\n")

print(levene_without_outliers)

# Kruskal-Wallis Test (Without Outliers)

kruskal_without_outliers <- kruskal.test(jump_total_distance_second_flash ~ Treatment, data = jump_data_total_distance_second_flash_clean)

cat("\n### Kruskal-Wallis test (Without Outliers)\n")

print(kruskal_without_outliers)

# Check for negative or zero values in 'Distance_moved_total'

if (any(jump_data_total_distance_second_flash_clean$jump_total_distance_second_flash <= 0)) {

min_value <- abs(min(jump_data_total_distance_second_flash_clean$jump_total_distance_second_flash, na.rm = TRUE))

jump_data_total_distance_second_flash_clean$jump_total_distance_second_flash <- jump_data_total_distance_second_flash_clean$jump_total_distance_second_flash + min_value + 0.00001

}

# Fit GLM (With and Without Outliers)

glm_without_outliers <- glm(jump_total_distance_second_flash ~ Treatment,

family = Gamma(link = "log"), data = jump_data_total_distance_second_flash_clean)

# Summary of GLM without outliers

cat("\n### GLM results (Without Outliers)\n")

summary(glm_without_outliers)

# EMMEANS and Pairwise Comparisons (Without Outliers)

cat("\n### EMMEANS and pairwise comparisons (Without Outliers)\n")

emmeans_without_outliers <- emmeans(glm_without_outliers, ~ Treatment, type = "response")

print(emmeans_without_outliers)

pairwise_without_outliers <- contrast(emmeans_without_outliers, method = "pairwise")

print(pairwise_without_outliers)

**# Code for experiment 2**

# Load necessary libraries

library(lme4) # Linear mixed models

library(lmerTest) # p-values in mixed models

library(emmeans) # Post-hoc comparisons

library(ggplot2) # Data visualization

library(readxl) # Read Excel files

library(dplyr)

library(tidyr)

library(car) # Levene's test and outlier detection

library(nortest) # Normality tests

# Convert relevant columns to appropriate types

data$Distance_total <- as.numeric(data$Distance_total)

data$Distance_zone_mean <- as.numeric(data$Distance_zone_mean)

data$Time <- factor(data$Time)

data$Treatment <- factor(data$Treatment)

# Assign Fish ID based on the Trial column

data_clean$Fish_ID <- as.numeric(factor(data_clean$Trial))

# Extract time in seconds from the 'Time' column

data_clean$seconds <- sapply(strsplit(as.character(data_clean$Time), "-"), function(x) {

time_parts <- as.numeric(unlist(strsplit(x[1], ":")))

return(time_parts[1] * 3600 + time_parts[2] * 60 + time_parts[3])

})

# Remove NA values from key variables

data_clean <- data_clean %>%

drop_na(Distance_total, seconds, Treatment)

# --- Ensure No Non-Positive Values ---

if (any(data_clean$Distance_total <= 0)) {

min_value <- abs(min(data_clean$Distance_total, na.rm = TRUE))

data_clean$Distance_total <- data_clean$Distance_total + min_value + 0.00001

}

if (any(data_clean$Distance_zone_mean <= 0)) {

min_value <- abs(min(data_clean$Distance_zone_mean, na.rm = TRUE))

data_clean$Distance_zone_mean <- data_clean$Distance_zone_mean + min_value + 0.00001

}

# --- Outlier Detection and Removal ---

# Identify outliers using standardized residuals from a linear model

outlier_test <- outlierTest(lm(Distance_total ~ Treatment, data = data_clean))

if (!is.null(outlier_test)) {

outliers <- as.numeric(names(outlier_test$rstudent))

data_clean <- data_clean[-outliers, ]

}

# --- Normality and Homogeneity Tests ---

# Shapiro-Wilk test for normality

shapiro_test <- shapiro.test(data_clean$Distance_total)

print(shapiro_test)

# Kolmogorov-Smirnov test for normality

ks_test <- ks.test(data_clean$Distance_total, "pnorm",

mean = mean(data_clean$Distance_total, na.rm = TRUE),

sd = sd(data_clean$Distance_total, na.rm = TRUE))

print(ks_test)

# Levene's test for homogeneity of variances

levene_test <- leveneTest(Distance_total ~ Treatment, data = data_clean)

print(levene_test)

# --- Statistical Analysis ---

# GLMM for total distance traveled

library(glmmTMB)

glmm_tmb_distance <- glmmTMB(

Distance_total ~ seconds * Treatment + (1 | Fish_ID),

family = Gamma(link = "log"),

data = data_clean

)

# Summary of the model

summary(glmm_tmb_distance)

# Post-hoc comparisons

posthoc_glmm_tmb <- emmeans(glmm_tmb_distance, pairwise ~ Treatment)

summary(posthoc_glmm_tmb$emmeans)

summary(posthoc_glmm_tmb$contrasts)

# --- Anxiety-Related Behavior Analysis ---

# GLMM for mean distance to the bottom

glmm_distance_zone <- glmmTMB(

Distance_zone_mean ~ seconds * Treatment + (1 | Fish_ID),

family = Gamma(link = "log"),

data = data_clean

)

# Summary of the model

summary(glmm_distance_zone)

# Post-hoc analysis for treatment effects on distance to bottom

posthoc_distance_zone <- emmeans(glmm_distance_zone, pairwise ~ Treatment)

summary(posthoc_distance_zone$emmeans)

summary(posthoc_distance_zone$contrasts)
